# Threefold reduction of modeled uncertainty in direct radiative effects over biomass burning regions by constraining absorbing aerosols

**DOI:** 10.1126/sciadv.adi3568

**Published:** 2023-12-01

**Authors:** Qirui Zhong, Nick Schutgens, Guido R. van der Werf, Toshihiko Takemura, Twan van Noije, Tero Mielonen, Ramiro Checa-Garcia, Ulrike Lohmann, Alf Kirkevåg, Dirk J. L. Olivié, Harri Kokkola, Hitoshi Matsui, Zak Kipling, Paul Ginoux, Philippe Le Sager, Samuel Rémy, Huisheng Bian, Mian Chin, Kai Zhang, Susanne E. Bauer, Kostas Tsigaridis

**Affiliations:** ^1^ Department of Earth Sciences, Vrije Universiteit, Amsterdam, Netherlands.; ^2^ Research Institute for Applied Mechanics, Kyushu University, Fukuoka, Japan.; ^3^ Royal Netherlands Meteorological Institute, De Bilt, Netherlands.; ^4^ Finnish Meteorological Institute, Kuopio, Finland.; ^5^ Laboratoire des Sciences du Climat et de l'Environnement, IPSL, Gif-sur-Yvette, France.; ^6^ European Centre for Medium-Range Weather Forecasts, Reading, UK.; ^7^ Institute for Atmospheric and Climate Science, ETH Zurich, Zurich, Switzerland.; ^8^ Norwegian Meteorological Institute, Oslo, Norway.; ^9^ Graduate School of Environmental Studies, Nagoya University, Nagoya, Japan.; ^10^ NOAA Geophysical Fluid Dynamics Laboratory, Princeton, NJ, USA.; ^11^ HYGEOS, Lille, France.; ^12^ Goddard Earth Sciences Technology and Research (GESTAR) II, University of Maryland at Baltimore County, Baltimore, MD, USA.; ^13^ NASA Goddard Space Flight Center, Greenbelt, MD, USA.; ^14^ Pacific Northwest National Laboratory, Richland, WA, USA.; ^15^ NASA Goddard Institute for Space Studies, New York City, NY, USA.; ^16^ Center for Climate Systems Research, Columbia University, New York City, NY, USA.

## Abstract

Absorbing aerosols emitted from biomass burning (BB) greatly affect the radiation balance, cloudiness, and circulation over tropical regions. Assessments of these impacts rely heavily on the modeled aerosol absorption from poorly constrained global models and thus exhibit large uncertainties. By combining the AeroCom model ensemble with satellite and in situ observations, we provide constraints on the aerosol absorption optical depth (AAOD) over the Amazon and Africa. Our approach enables identification of error contributions from emission, lifetime, and MAC (mass absorption coefficient) per model, with MAC and emission dominating the AAOD errors over Amazon and Africa, respectively. In addition to primary emissions, our analysis suggests substantial formation of secondary organic aerosols over the Amazon but not over Africa. Furthermore, we find that differences in direct aerosol radiative effects between models decrease by threefold over the BB source and outflow regions after correcting the identified errors. This highlights the potential to greatly reduce the uncertainty in the most uncertain radiative forcing agent.

## Teaser

Constraining absorbing biomass burning aerosols greatly reduce the uncertainty of regional aerosol direct radiative effects.

## INTRODUCTION

Biomass burning (BB) is a leading contributor to global emissions of carbonaceous aerosols that can potentially exacerbate climate warming by absorbing solar radiation at visible to ultraviolet (UV) wavelengths [i.e., black carbon (BC) and organic aerosol (OA), the absorbing component of OA is also referred to as brown carbon] (*1*, *2*). Therefore, these absorbing BB aerosols (BBAs) can essentially affect radiation balance (*2*, *3*), cloud formation and properties (*4*, *5*), precipitation (*6*, *7*), and regional circulation patterns (*8*, *9*). To better understand these impacts, recent flight campaigns have paid particular attention to the BBA over tropical regions where large amounts of BBA are injected into the atmosphere every year (*10*–*13*).

Despite the recent progress in airborne measurements of BBA, large-scale and long-term assessments of the climate impacts of BBA have far relied on global aerosol models (*14*, *15*). However, models have shown substantial discrepancies and errors regarding BBA absorption (*16*–*19*), which remains a key obstacle to reliable climate assessments because BBA absorption can simultaneously contribute to direct, semidirect, and indirect aerosol effects (*9*, *20*). Comparisons between models and satellite observations suggest that global models generally underestimate the overall warming impacts of BBA, as indicated by a substantial underestimation of the aerosol absorption optical depth (AAOD) (*21*) in combination with an overestimation of the single-scattering albedo (SSA) (*20*). These findings are supported by flight campaign measurements (*22*, *23*) such as the ObseRvations of Aerosols above CLouds and their intEractionS (ORACLES) project (*22*), suggesting that the overall warming caused by BBA plumes is greater than previously considered, particularly in tropical regions. However, when considering the aerosol composition, global models tend to produce stronger absorption than observed from field and laboratory measurements for a given BC mass mixing ratio (*24*). The contradiction between the underestimated overall warming and overestimated absorbing capability per unit of BC mass highlights important errors in the emission, composition, and optical properties of absorbing BBA. Although there have been discussions regarding the possible reasons for these model errors [e.g., particle size distribution, vertical profiles, mixing states, and refractive index (*24*–*28*)], a quantitative evaluation of how these factors contribute to the overall errors is still missing. This poses a fundamental challenge to the aerosol modeling community, which hinders reliable climate assessments over tropical regions.

Here, we investigate the seasonal- and regional-scale AAOD over two key BBA-emitting areas in the world (the larger Amazon region and Southern Africa; see fig. S1) by breaking AAOD down into three factors

AAOD=E×τ×MAC (1)

where *E*, τ, and MAC denote seasonal carbonaceous aerosol (BC + OA) emissions, aerosol lifetime, and mass absorption coefficient (MAC), respectively. Other absorbing components (e.g., dust) account for only 4% of AAOD in the model ensemble over the two focused regions and are therefore ignored. Because of the definitions of aerosol lifetime (column burden of BC + OA divided by *E*) and MAC (AAOD divided by the column burden of BC + OA), this equation is always applicable. Both lifetime and MAC are emergent properties of models, resulting from various physical and chemical processes. MAC has been identified as an important but highly uncertain parameter (*29*) and exhibits a wide range across models (*19*). Note that we consider secondary OA (SOA) formation as part of the total emissions given the short formation time scale compared to the seasonal time scale in our analysis (*30*).

**Fig. 1.**
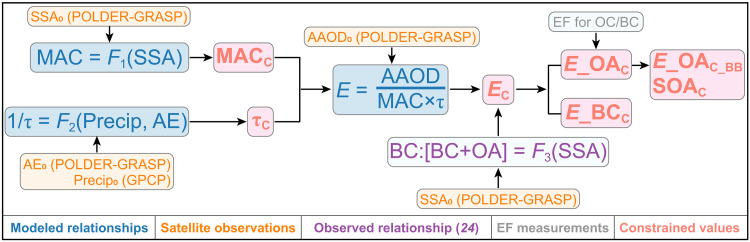
Flowchart showing the procedures of the constraining analysis in the current study. The colors indicate the modeled relationships (blue), satellite observations (orange), in situ relationships (purple), field measurements of emission factors (EFs) for the primary BBA (gray), and constrained estimates (red). In particular, the satellite observation is labeled with a subscript of “0,” and constrained values are labeled with a subscript of “C.” *F*_1_ and *F*_2_ show the linear regressions developed from the AeroCom models. *F*_3_ indicates the observed relationship from (*24*). Abbreviations/notations are defined as follows: SSA (single-scattering albedo), AE (Angstrom Exponent), Precip (precipitation), MAC (mass absorption coefficient), τ (lifetime), and *E* (emission).

The overall procedure for constraining the above three components is somewhat similar to strategies commonly used in the context of “emergent constraints,” but we have introduced a closure relation (based on a simple box model) that allows estimating three components from two modeled relationships. It is similar to the methodology applied in a previous study (*31*), in which we analyze AOD and considered all aerosols instead of carbonaceous aerosols only. Briefly, we linearly regressed modeled MAC against modeled SSA and modeled τ against modeled precipitation and the angstrom exponent (AE; an indicator of ambient particle size) using the model data from the AeroCom (Aerosol Comparisons between Observations and Models; see table S1) project. Then, we applied satellite observations of SSA to estimate the constrained MAC and similarly constrain τ from observations of precipitation and AE. Last, we used Eq. 1 to constrain *E*. The constrained values of *E*, τ, and MAC allow us to attribute AAOD errors to contributions from these three factors for individual models. Uncertainty analysis is conducted for all these constraining processes as shown in Fig. 1. Notably, the constrained results are only effective on a regional and seasonal scale, and caution must be exercised when directly applying these results to smaller scales. This work presents advancements upon the foundation of (*31*) as it constrains MAC instead of MEC (mass extinction coefficient). In addition, we implement in situ data in the interpretation of satellite observations that allows a disaggregation of BC and OC emissions. Furthermore, in our seasonal, regional analysis, we find that SOA formation is important for fire aerosols over the Amazon but not over Africa.

## RESULTS

### Constraining total emission, lifetime, and MAC

**Fig. 2.**
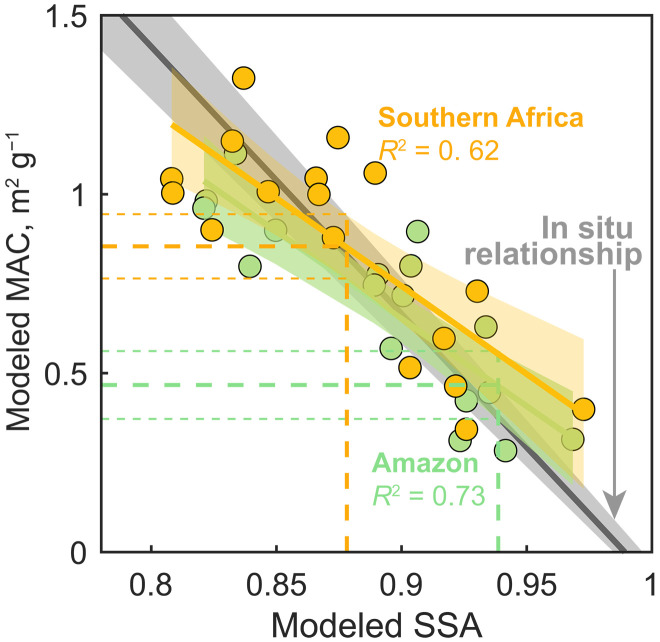
Relationships between modeled MAC and modeled SSA in the Amazon and Southern Africa. Each dot represents the seasonally averaged data from a single model, with colors indicating the two fire regions. MAC is calculated as AAOD/(BC + OA). SSA is for total aerosols. The solid color lines indicate the linear regressions with 95% confidence intervals (shaded areas). Vertical dashed lines denote the regional SSA observations from satellite (see Materials and Methods), and the horizontal dashed lines show the constrained MAC with 95% confidence intervals (horizontal dotted lines). The in situ relationship (*24*) is shown as the mean (black solid line) and 95% confidence interval (gray shaded area). Note that the in situ relationship is built for aerosols with a high carbonaceous content (≥85% of the total aerosol mass). For AeroCom models, we show that the fire-season averaged SSA well represents such a conditional SSA for BBA given the prevailing abundance of carbonaceous aerosols (see Materials and Methods).

Despite the substantial variation in MAC values in the AeroCom models, we find a linear relationship between the modeled MAC and SSA (Fig. 2); both variables depend strongly on the BC mass mixing ratio within the total aerosols. Such a linear relationship compares favorably with in situ and laboratory observations (Fig. 2) (*24*). The relationship is also confirmed by Mie calculations with varying configurations (e.g., mixing state, refractive index, and particle size; fig. S2). This suggests that the modeled relationship between MAC and SSA in AeroCom is robust. We then combine satellite observation of SSA with the linear relationship to constrain the MAC.

Similar to constraining MAC, we constrain aerosol lifetime via linear regression with precipitation and AE, as derived from AeroCom models and the satellite observations of the two predictors (fig. S3 and Materials and Methods). This relationship can be expected because precipitation and particle size affect aerosol removal, with a dominant impact from precipitation over our study regions (*31*, *32*). AeroCom mean precipitation patterns agree very well with observations (see the correlations; fig. S4), suggesting that AeroCom mean wet deposition patterns are realistic.

Equation 1 enables an estimate of carbonaceous aerosol emissions from satellite observations of AAOD and with constrained values of MAC and aerosol lifetime. Given that the carbonaceous component is the dominant contributor to the total aerosol emissions over the study regions, we compare the constrained emissions with the total emissions (for both carbonaceous and other species) from our previous work using independent AOD and aerosol extinction data (*31*) and find that these are in good agreement (fig. S5).

**Fig. 3.**
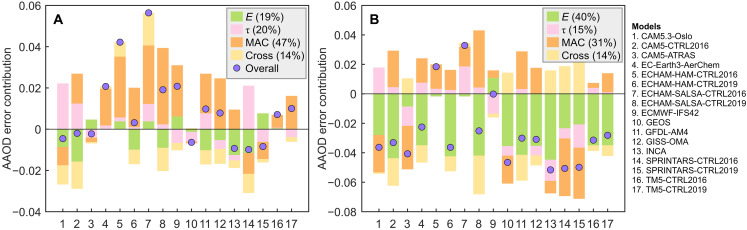
Absolute AAOD errors due to individual components in AeroCom models. Results are shown individually for the Amazon (**A**) and Southern Africa (**B**). The calculation of the errors due to emission (*E*), aerosol lifetime (τ), MAC, and cross terms (Cross) is described in the Materials and Methods. The numbers in the legend indicate the average contribution of each component for all the AeroCom models.

The constrained values for the three factors considered in Eq. 1 allow us to investigate the AAOD errors due to each factor’s contribution in the AeroCom models. As shown in Fig. 3, we find that the contributions of the three factors to the overall AAOD errors for each model are diverse and exhibit substantial compensation. Over the Amazon, the AAOD errors mainly arise from biased MAC (47 ± 24%). In contrast, the AAOD errors in Africa are more related to underestimated emissions in the models (40 ± 20%), although the MAC contribution is also important (31 ± 16%). In comparison, the contribution from lifetime error is smaller. This suggests the importance of correcting the MAC errors (or errors in SSA, as discussed in text S1 and figs. S2 and S6) and emission errors in models to improve the overall performance.

To verify the reliability of the constrained results, we predict the African outflow AAOD for the AeroCom models through a metamodel analysis (see text S2). We show that adopting the constrained results (*E*, τ, and MAC) allows an accurate prediction of the AAOD over the African outflow region (fig. S7), providing independent confirmation on the above constraining analysis and error attribution.

### BC and OA emissions in BB regions

**Fig. 4.**
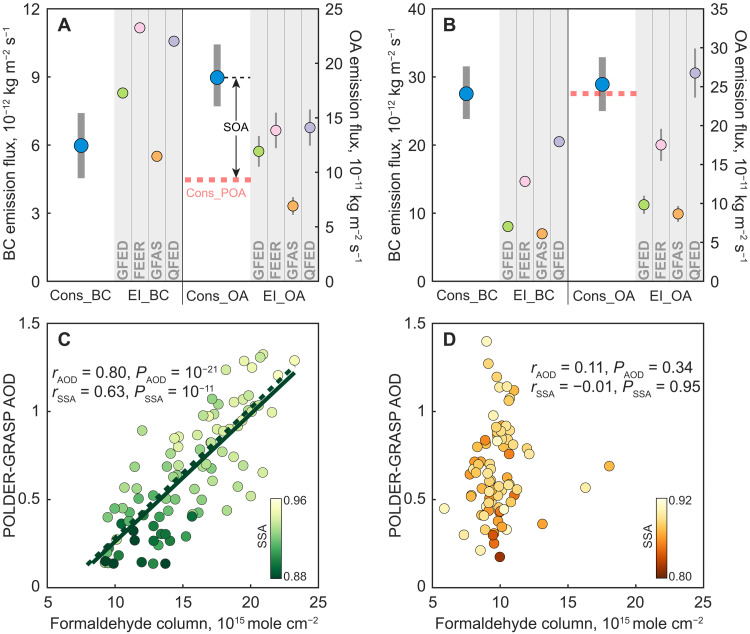
The constrained emissions for BC and total OA. The constrained emissions (Cons_BC, Cons_OA) over the Amazon (**A**) and Southern Africa (**B**) are shown with interquartile ranges (blue dots with thick error bars). Note that Cons_OA indicates total OA, which is further separated into primary BB emission (Cons_POA) and SOA formation (SOA). The constrained results are compared with four BB emission inventories (EI_BC and EI_OA). The thin error bars for EI_OA indicate the ranges of the OA/OC ratio ranging from 1.5 to 1.9, with the dot showing the mean emission (i.e., OA/OC = 1.7). Bottom: Correlations between satellite AOD and formaldehyde column for the Amazon (**C**) and Southern Africa (**D**) to support our constrained SOA, given that formaldehyde is an indicator of SOA formation. The AOD and SSA data are from Polarization and Directionality of the Earth’s Reflectances with the Generalized Retrieval of Aerosol and Surface Properties algorithm (POLDER-GRASP), and the formaldehyde column is from Ozone Monitoring Instrument (OMI). Both satellite products are collocated with each other at 1° × 1° × daily grid cells during fire seasons. Each dot represents the daily average of AOD and the formaldehyde column after collocation. The color scale denotes the SSA observation. Correlations between formaldehyde and AOD (*r*_AOD_) and between formaldehyde and SSA (*r*_SSA_) are shown with *P* values. In (C), the solid line indicates the regression based on the data points as shown. The dashed line is a regression derived from in situ measurements of formaldehyde and OC (*45*), here converted to formaldehyde column and AOD, assuming similar vertical profiles and an MEC of 5.9 m^2^ g^−1^ (*31*).

Although the above analysis highlights the errors in total emissions, it can provide more valuable insights if the total emissions can be speciated into either BC or OA emissions with a constrained rBC [i.e., BC:(BC + OA)]. To achieve this goal, we use the linear relationship observed by (*24*) between rBC and SSA to estimate ambient rBC from satellite observations of SSA. AeroCom models allow us to establish a relationship between ambient and emitted rBC, which, in any case, is close to identical (see Materials and Methods). These constrained BC and OA emissions can be compared with four widely used inventories of BC and OC emissions [i.e., Global Fire Emission Database version 4.1s (GFED), Global Fire Assimilation System version 1.2 (GFAS), Fire Energetics and Emission Research version 1.2 (FEER), and Quick Fire Emissions Dataset version 2.5 (QFED)], providing that we have reasonable OA/OC ratios (Fig. 4). Field measurements of primary BBA emissions exhibit a narrow range (1.5 to 1.9) for OA/OC (*33*–*39*). We find that most inventories estimate lower emissions than our estimates over Africa, which partly explains the negative AAOD bias (Fig. 3B), as these inventories are used in AeroCom models (table S1). Inventories with underestimated emissions may be related to undetected small fires (*40*). The discrepancy is also affected by the variation in emission factor (EF), but it is unlikely to fully explain the low emissions, as the EFs used in the inventories generally agree with field measurements (see table S2 and fig. S8). In the Amazon, the inventories also estimate lower OA emission levels relative to our constrained OA. However, the BC inventory emissions are generally higher (except in the GFAS inventory), which is consistent with the overestimated MAC in models (Fig. 3A). Again, this cannot be explained by uncertainties in the EFs (fig. S8), which implies that the low OA emissions in the inventories over the Amazon result from missing sources.

It is important to mention that the AeroCom models also apply OA/OC ratios to estimate OA emissions from the inventories. However, their OA/OC ratios (1.4 to 2.6; see table S1) tend to spread larger than the aforementioned field measurements, suggesting that part of the emission errors shown in Fig. 3 are due to inappropriate OA/OC ratios.

### Contribution of SOA formation to total OA

Our constrained OA emissions consist of both primary emission and SOA formation, which can be a potential reason for the differences between our results and the inventories. Because all BB BC results from primary emissions, we constrain primary OA emissions (POA) with in situ measurements of OC/BC for the BB source (R_OC/BC_; see table S2) and an assumed OA/OC ratio of 1.7 (see Fig. 4), as shown in Eq. 2

$$\mathrm{POA}={\mathrm{BC}}_{C}\times R_{\mathrm{OC}/\mathrm{BC}}\times 1.7$$ (2)

where BC_C_ denotes the total constrained BC emission. Given the prevalence of tropical forest and deforestation fires in the Amazon and savanna fires in Africa (fig. S9), we use the average EF values corresponding to each fire type to compute OC/BC for the respective regions. Over the Amazon, the constrained POA is much lower than the total OA emissions, suggesting a substantial contribution from SOA. According to the difference between constrained POA and total OA emissions, we estimate that SOA formation accounts for 52% of total OA emissions over the Amazon. Such a contribution is comparable to studies based on in situ observations (*41*). The estimated SOA formation is affected by the choice of OA/OC ratio for primary OA emissions, but it does not fundamentally alter the large contribution (47 to 58% with OA/OC ranging from 1.5 to 1.9). Uncertainty in the OC/BC ratio also affects the SOA contribution, but we estimate its contribution to range from 45 to 58% (interquartile range; see Materials and Methods). Likewise, using an OC/BC ratio weighted by fire type (tropical forest and savanna) does not substantially alter our results. In contrast with the Amazon, we find that SOA formation contributes little over Southern Africa, as revealed by the small difference between constrained total and primary OA emissions (Fig. 4B).

Support for this somewhat unexpected finding comes from the correlation between AOD and formaldehyde columns derived from satellite observations (Fig. 4, C and D). Formaldehyde has a very short lifetime and shares the same chemical pathway as other low-volatility species (*42*). Consequently, it has been interpreted as an indicator of SOA formation (*43*). Although fires also emit formaldehyde, observed quantities on a regional scale are much too high to be explained in that way (*44*). Over the Amazon, formaldehyde correlates with both AOD and SSA, suggesting primarily an increase in non- or low-absorbing aerosols with higher formaldehyde columns (or more active SOA formation). In situ observations of OA and formaldehyde in smoke plumes (*45*) agree with our results (Fig. 4C), confirming the validity of the satellite measurements. Differently, there is no significant correlation between AOD and formaldehyde over Africa (*P* > 0.1; Fig. 4D), consistent with the aforementioned low SOA contribution. Our results highlight the potential of detecting and constraining SOA formation from aerosol absorption.

### Correcting AAOD errors in two global models

**Fig. 5.**
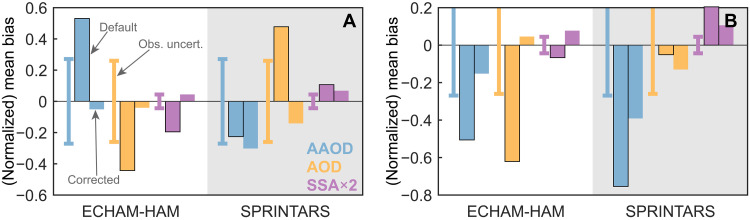
Seasonal mean modeling errors for default and corrected simulations in global models. Results are shown for ECHAM-HAM and SPRINTARS models over the Amazon (**A**) and Southern Africa (**B**). The two global models produce the most absorbing (ECHAM-HAM) and most scattering aerosols (SPRINTARS) in the AeroCom model ensemble (see fig. S6). The target variables include AAOD, AOD, and SSA. The normalized mean bias for AAOD and AOD and the mean bias of SSA (doubled to match axis scale) are shown for the whole fire season. Two configurations are considered for each model, including the default (bars with solid edges) and corrected simulations (bars without solid edges) based on constrained aerosol properties. Details of the model settings can be found in Materials and Methods and table S2. All the model data are collocated and validated with POLDER-GRASP. The vertical solid lines indicate the observation uncertainties of AAOD, AOD, and SSA for POLDER-GRASP. The observation uncertainty is calculated as the average of the absolute errors for POLDER-GRASP compared with AERONET sites, as shown in fig. S1. The POLDER-GRASP and AERONET datasets are collocated with each other before calculating these observation uncertainties.

The above error analysis is used to improve two global models by correcting the identified errors in the three components (Materials and Methods and table S3). The ECHAM-HAM [a model developed from the atmospheric model by European Center for Medium-Range Weather Forecasts (EC) and a parameterization package developed at Hamburg (HAM)] and SPRINTARS (Spectral Radiation-Transport Model for Aerosol Species) models are selected given their opposite SSA errors in the AeroCom ensemble (fig. S6). The models have been thoroughly validated against satellite observations, which inherently have a considerable retrieval uncertainty in AAOD data. However, it is worth noting that this retrieval uncertainty is generally smaller than the default errors in the model. As shown in Fig. 5, our corrections over the BBA source regions in both models have reduced the seasonal AAOD error, especially in the ECHAM-HAM model. Although the SPRINTARS AAOD error has slightly increased following the corrections, it remains comparable to the uncertainty associated with satellite retrievals. The SSA is also found to better agree with satellite observations, suggesting the robustness of our analysis and corrections. In addition, the modeled AOD exhibits much smaller errors, which independently verifies our analysis. Moreover, our corrections over the source regions also benefit the simulations in the outflow areas, with smaller errors found for AAOD, AOD, and SSA in the two models (figs. S10 and S11). This model improvement is also found when validated against independent Aerosol Robotic Network (AERONET) observations (fig. S12).

**Fig. 6.**
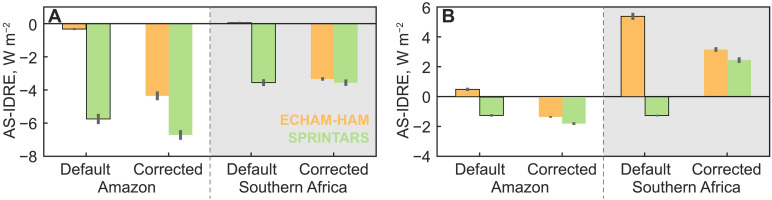
Modeled all-sky instantaneous direct radiative effect (AS-IDRE) on top of the atmosphere. The results are shown for ECHAM-HAM (orange) and SPRINTARS models (green) over the Amazon and Southern Africa source (**A**) and outflow regions (**B**). Data from the default and corrected simulations are shown as bars with and without edges, respectively. The IDRE is averaged for all fire seasons and regions, with error bars indicating the SEs of the daily variation during fire seasons.

As a result of the corrections, the difference in the all-sky instantaneous direct radiative effect (IDRE) between the two models is greatly reduced. By default, the SPRINTARS model produces much stronger cooling effects than the ECHAM-HAM model over the two BB source regions (Fig. 6 and fig. S13). This difference becomes much smaller after our correction. In previous studies, the spread of multiple models has been used to characterize the uncertainty of aerosol climate impacts [e.g., (*46*)]. Assuming the difference between ECHAM-HAM and SPRINTARS IDRE as an indication of IDRE uncertainty for the AeroCom ensemble, our correction reduces such an uncertainty by 71% (from 4.5 to 1.3 W m^–2^; see table S4). The correction results in a stronger reduction in Africa for the IDRE difference between the two models, mostly because of the higher BC content in African aerosols. In addition, an even larger reduction (by 86%, from 4.2 to 0.6 W m^–2^) of the IDRE difference is observed over the outflow regions, where the default models produce IDREs with opposite signs (fig. S13). In particular, the default SPRINTARS model simulates a cooling effect in the African outflow region, which differed fundamentally from the overall warming effect as reported from aircraft measurements (*15*). The African outflow region has large areas covered by quasi-permanent stratocumulus clouds with bright surfaces, which amplifies the change in IDRE due to our modification of absorbing aerosols above the cloud deck. This explains the larger changes in the IDRE differences in the African outflow region than in the source region. Moreover, our modifications have obtained a better agreement between the two models for the IDRE patterns over both the African source and outflow regions, with the spatial correlations increasing from 0.27 to 0.73. Note that the all-sky IDRE is also affected by the different cloudiness in the two models. Our results suggest a potential for constraining the model uncertainty from the aerosol perspective.

## DISCUSSION

In this study, we constrain the aerosol absorption over two tropical BB regions (the Amazon and Southern Africa) through a combination of AeroCom models and in situ and satellite observations. Errors in three key AAOD factors (emission, lifetime, and MAC) are quantified, and we identify leading contributions from emission and MAC to total AAOD errors in the AeroCom models. Corrections for these identified errors bring two models more in line with the observations, confirming the reliability of our analysis. The corrections reduce the modeled uncertainty in the direct radiative effects by 71% (from 4.5 to 1.3 W m^–2^) over the BB source regions, with an even larger reduction in the outflow areas (by 86%, from 4.2 to 0.6 W m^–2^). This indicates the potential of our methodology to greatly reduce the overall uncertainty of the aerosol climate impacts. The results also suggest the necessity of dealing with errors in refractive index, particle size distribution, and precipitation in models for future model development.

In addition to primary BB emissions, our analysis suggests that substantial SOA formation contributes to the modeled aerosol absorption errors over the Amazon but not over Africa. The remarkable regional difference may be associated with the variation in precursor gas emissions from biogenic sources, with a notably higher level of such emissions found over the Amazon than over Africa (fig. S14). In comparison, model estimates of SOA production are very uncertain and usually fail to show regional differences between the Amazon and Africa. Our estimation, to our knowledge, is the first one in which SOA formation is constrained by aerosol absorption, which provides an innovative perspective on SOA estimation. The results also highlight the importance of SOA during fire seasons. This particular aspect has often been overlooked, and our findings emphasize the need for further research.

Our work presents an integration of satellite and in situ observations, providing a more comprehensive and robust conclusion than relying on a single data source. Our work rigorously considers uncertainties in these observations. In particular, the satellite-derived aerosol absorption retrieval errors contribute the most to the overall uncertainty in this study (fig. S15), even when using the most reliable satellite products. This suggests that the impending improvement of satellite products will greatly reduce the uncertainty and promote the utility of our method.

## MATERIALS AND METHODS

### Models and variables over fire regions

We base our analysis on data from 17 AeroCom models, collected from two control experiments conducted in 2016 (CTRL2016) and 2019 (CTL2019). Both experiments perform model simulations for 2010 with default model configurations (table S1). The selected variables from these models include AOD at 440 and 550 nm, AAOD at 550 nm, emissions, column burdens, and precipitation. AE (550 versus 440 nm), SSA (550 nm), and MAC (550 nm) are calculated from these fields. We focus our analysis on two major BB emission regions, the Amazon and Southern Africa (see fig. S1), as defined by model emissions. We consider the data during fire seasons only (July to October and June to September for Amazon and Southern Africa, respectively).

### Observation data

Previous studies have suggested large and diverse errors in the satellite-based observations of AAOD and SSA (*47*). To characterize satellite errors, we consider three datasets: the Polarization and Directionality of the Earth’s Reflectances with the Generalized Retrieval of Aerosol and Surface Properties (POLDER-GRASP) algorithm, the Ozone Monitoring Instrument (OMI) with UV aerosol algorithm (OMAERUV), and the Advanced Along-Track Scanning Radiometer with Optimal Retrieval of Aerosol and Cloud algorithm (AATSR-ORAC). We validate the three datasets against AERONET data (the locations of the AERONET sites used in this study are shown in fig. S1) following the procedure in (*47*) and find that POLDER-GRASP exhibits the lowest error and has the highest correlations with AERONET data for both AAOD and SSA (fig. S16). Therefore, we use POLDER-GRASP as the satellite observation (including AE) during 2010 fire seasons throughout the analysis. However, even for POLDER-GRASP, large retrieval errors exist, which contribute substantially to the overall uncertainties presented in this work.

To apply Eq. 1 in our analysis, a regional estimation of AAOD is required, which cannot be obtained directly from the sparsely sampled raw satellite data. Following the homogenization method in our previous work (*31*), we perform a linear regression between the modeled regional AAOD and modeled AAOD with POLDER-GRASP sampling (averaged over the region and fire season; fig. S17). The raw POLDER-GRASP data are then applied to the regression to estimate the regional AAOD. A similar method is also used to estimate the regional SSA. The robustness of the method is verified through a jackknife test by removing the models one by one, which produces small relative variations in the predicted regional AAOD and SSA (<1%), suggesting that the construction of regional values is independent of the models used. The regional observations of AAOD and SSA are also used to validate the models on a seasonal scale (see fig. S18), showing a varying degree of error per model. Broadly, models tend to underestimate SSA over the Amazon (by 0.05 on average) and underestimate AAOD over Southern Africa (by 32% on average).

In addition to aerosol observations, daily precipitation data are taken from the global precipitation climatology project (GPCP), which has been proven to be superior to other reanalysis datasets (*48*). The formaldehyde column is obtained from the OMI onboard the Aura satellite (*49*). Field measurements of fire EFs are also collected for the prevailing tropical forest/deforestation fires in the Amazon and the savanna/grassland fires in Southern Africa (table S2). All these observations are collected for the fire seasons during 2010.

### Constraining BB carbonaceous aerosols in models

The overall constraining procedure is displayed in Fig. 1. The basic idea is to decompose AAOD into three interpretable factors: emission, lifetime, and MAC (see Eq. 1). The emission is calculated as the total of the BC and OA emissions; lifetime is calculated as the (burden of BC + OA)/(emission of BC + OA), and MAC is AAOD/(burden of BC + OA). All the variables are calculated as regional fire-season averages. Other absorbing components (e.g., dust) are assumed to have negligible impacts given the small contribution indicated by the observations (*50*) and AeroCom models (4%) for the two regions. In addition, we find a small absorption AE from POLDER-GRASP observations (fig. S19), suggesting that brown carbon is not important (*51*, *52*). Note that the emission in Eq. 1 includes both primary emissions and those from secondary formation (SOA), as the latter happens on a much smaller time scale than the seasonal average that we are working with.

The constrained aerosol lifetime and MAC are predicted by applying observations [of precipitation (Pr) and AE and SSA, respectively] to the following linear regressions built from the AeroCom models

1/τ=αPr+βAE+A;MAC=γSSA+B (3)

We have also tried to include other predictors in the regressions (e.g., plume height), which cannot improve the performance of regressions. With the constrained aerosol lifetime and MAC, we estimate the total emission based on the regional observation of AAOD. These constrained values allow us to attribute the modeled AAOD errors due to total emission (∆AAOD*_E_*), aerosol lifetime (∆AAOD_τ_), MAC (∆AAOD_MAC_), and cross terms (*C*), as described in Eq. 4

$$\begin{array}{c} {\mathrm{AAOD}}_{C}+\text{Δ}\mathrm{AAOD}=(E_{C}+\text{Δ}E)(\tau_{C}+\text{Δ}\tau )({\mathrm{MAC}}_{C}+\text{Δ}\mathrm{MAC})\\ \text{Δ}\mathrm{AAOD}=\text{Δ}E\tau_{C}\;{\mathrm{MAC}}_{C}+E_{C}\;\text{Δ}\tau {\mathrm{MAC}}_{C}+E_{C}\tau_{C}\text{Δ}\mathrm{MAC}+C\\ =\text{Δ}{\mathrm{AAOD}}_{E}+\text{Δ}{\mathrm{AAOD}}_{\tau }+\text{Δ}{\mathrm{AAOD}}_{\mathrm{MAC}}+C \end{array}$$ (4)

where the “∆” and subscript “C” indicate the modeled errors and constrained values, respectively.

In addition to the total emissions for BC + OA, the rBC ratio is constrained in this work by using the observational relationship between SSA and ambient rBC at 550 nm wavelength from (*24*). Here, we do not use the modeled relationship between rBC and SSA, as we find that it contains a large error (see fig. S6). It should be noted that the relationship by (*24*) was established under the criteria that OA and BC accounted for more than 85% of the total aerosol mass (to focus on BBAs). For the AeroCom models, we compare the SSA for total aerosols and the SSA of grid cells with ≥85% carbonaceous aerosol components, with small differences being found for most models, especially within the range of the SSA observations (see fig. S20A). This suggests that the SSA for total aerosols during fire seasons could sufficiently represent the aerosol criteria by (*24*), which allows us to constrain the rBC in the ambient aerosols from SSA on the basis of the observed relationship. Using another regression to link the rBC from ambient aerosols to the emissions (see fig. S20B), we lastly obtain the constrained rBC in emissions and estimate the separate emissions for BC and OA.

To assess the robustness of our constraining procedure, we use individual AeroCom models to serve as a truth and generate perfect (i.e., errorless) synthetic observations from it. The remaining models are then used to predict the emission, lifetime, and MAC of the truth run following the same procedure as described above (the regression between SSA and ambient rBC is replaced with the modeled relationship; see fig. S6). As shown in fig. S21, the predicted values agree well with truth model data, demonstrating the robustness of the methodology. In particular, the average of the absolute relative errors for predicted BC emissions are 17 and 14% for the Amazon and Southern Africa, respectively, which are much smaller than the uncertainty in our main analysis using real observations. This suggests that the overall uncertainty of our constraining analysis is primarily affected by observational errors, as corroborated by our uncertainty analysis shown in fig. S15.

### Uncertainties of the constraining analysis

We consider three types of uncertainty in our constraining analysis: (i) Satellite retrieval uncertainties for AAOD, SSA, AE, and precipitation. The first three are set as 27%, 0.022, and 0.23 according to validations with AERONET over the studied domain, and the last is set as 9% according to the error in GPCP (*53*); (ii) the uncertainties for estimating regional values (AAOD, SSA, and AE; see fig. S17); and (iii) the uncertainty in constraining lifetime, MAC, and emissions for BC and OA (see Eq. 2) due to the uncertainty in the regressions developed from the AeroCom models (Fig. 2 and figs. S3 and S20B) and from (*24*) (see fig. S6). We use a Monte Carlo method to estimate the overall uncertainties by repeating the constraining analysis 100,000 times by randomly dropping a data point of each input from the distributions built from their stated uncertainties. The interquartile range of the results is used to characterize the uncertainty in this study. Uncertainties due to individual factors are also estimated. For the constrained BC and OA emissions, the satellite retrieval errors of AAOD and SSA contribute the most to our constrained values (fig. S15).

To account for the uncertainty of speciation between POA and SOA, we investigate the impacts of variations in EFs across in situ studies. This is achieved by constructing normal distributions for BC and OC using the means and SEs for their EFs based on individual studies (table S2). Subsequently, we perform random draws from these distributions to estimate the resulting variations in OC/BC. Upon inclusion of these uncertainties, the estimated SOA contribution spans from 45 to 58% (as interquartile) over the Amazon and 0 to 14% over Africa. We also investigate the uncertainty by assuming log-normal distributions for EFs. The resulting variations in SOA contribution expand to a range of 49 to 62% over the Amazon and from 0 to 10% over Africa. Moreover, as both regions encompass multiple fire types, we have also used GFED data to obtain a regional average OC/BC weighted by fire type. Consequently, the estimated SOA contribution is 58% (53 to 62% as interquartile) over the Amazon, slightly higher than the previously mentioned value (52%; 45 to 58%) but still well within the associated uncertainty range. In Africa, a low SOA contribution of 1% is computed. These results suggest that incorporating variations in EFs would not alter our SOA estimation fundamentally, lending further support to the robustness of our SOA assessments.

### Global model simulations and corrections

We conduct simulations in two global models that produce the most negative (ECHAM-HAM) and positive (SPRINTARS) SSA errors in the AeroCom ensemble (fig. S6). The latest version of ECHAM-HAM (ECHAM6.3.0-HAMMOZ2.3) is run at a T63 horizontal resolution (~1.875°) and 47 vertical level (*54*). The SPRINTARS model presents simulations at the T213 horizontal grid (0.5625°) with 40 vertical hybrid layers (*55*). Both simulations start in January 2010 with runs before the fire seasons as spin-up, and validations are made during the fire seasons. The ECHAM-HAM model includes inactive SOA following the prescribed emissions in (*56*), while SPRINTARS calculates the oxidation of precursors (terpene and isoprene) at a prescribed emission level over land according to the Global Emissions Initiative dataset. With default configurations, the differences in modeled SSA and direct radiative effects between two models are likely associated with the very different particle size distribution and refractive index, the two factors that affect SSA the most in the AeroCom models (text S1). Accordingly, we modify the modeled particle size and BC refractive index in the two models. In addition, emission and lifetime are corrected on the basis of our constrained results (table S3). The detailed corrections are listed below for MAC (1 and 2), lifetime (1 and 3), and emission (4).

1) Particle size. The modeled ambient particle size is modified to match the observed AE. For ECHAM-HAM, we increase the emitted/ambient particle size by referring to our previous study (*31*). In SPRINTARS, we switch off the hygroscopic growth for OA which is likely too strong (*57*). Modifications to both models produce better agreement with the AE observations from POLDER-GRASP (fig. S22A). The AE bias in the default simulations and the corresponding improvement through corrections are further supported by AERONET observations (fig. S22B).

2) Refractive index for BC. According to field measurements (*58*, *59*), the imaginary part of refractive index of BC in the two models is changed to 0.3*i*. Note that this value is lower than those used in the AeroCom models (0.44 to 0.79 for the imaginary part; see table S1). We conduct a sensitivity test on the imaginary part ranging from 0.1 to 0.5 with the corrected ECHAM-HAM model and find that the modified particle size with a value of 0.3 agrees the best with SSA observations from (*24*), suggesting that the observation-based refractive index is more suitable than those used in the AeroCom models (see fig. S23). In addition to SSA, this correction also results in better agreement with our constrained MAC for the two models (fig. S24). This refractive index is also used in a previous model study (*60*). Moreover, we also test different real parts of refractive index by changing values from 1.4 to 1.95 encompassing both observed values (*61*) and model-recommended values (see table S1). The resulting changes in AAOD are negligible (<1%), and we maintain the default model values.

3) Precipitation. A scaling factor is directly added to the modeled wet deposition based on the default precipitation error. This will correct the lifetime together with the modified particle size as stated above.

4) Emissions. BC and total OA emissions (both primary emissions and those from secondary formation) are scaled to our constrained results.

Details of the parameterizations can be found in table S3, and the impacts of the modifications on SSA and MAC are shown in fig. S24. The corrected simulations are validated against POLDER-GRASP (Fig. 5 and figs. S10 and S11), showing better agreement than the default simulations. Please note that the above modifications are conducted specifically over the selected regions during fire seasons and may not be directly applicable to different times or domains.

In addition to the simulations for 2010, we extend our constraining analysis and model correction for the year 2009, which shows a lower AAOD observation than 2010 (−47% for the Amazon and −10% for Africa). This assumes that the modeled relationships established from 2010 data are applicable to a different year, enabling us to use the 2009 observation data to constrain the models. Such an assumption is supported by the substantial model improvement after correction for 2009 (fig. S25). This compelling result underscores the robustness of our methodology, as it is independent of the specific year of model data chosen for analysis.

## Supplementary Materials

### This PDF file includes:

Supplementary Text S1 and S2 Figs. S1 to S25 Tables S1 to S4 References
