# Supplementary material for: Threefold reduction of modeled uncertainty in direct radiative effects over biomass burning regions by constraining absorbing aerosols
Source: Sci Adv. 2023 Dec 1;9(48):eadi3568. doi: 10.1126/sciadv.adi3568 (PMC10691779; doi:10.1126/sciadv.adi3568)
Supplement: Supplementary file 1 — Supplementary Text S1 and S2 Figs. S1 to S25 Tables S1 to S4 References [file sciadv.adi3568_sm.pdf]

Science Advances
AAAS

Supplementary Materials for

Threefold reduction of modeled uncertainty in direct radiative effects over biomass burning regions by constraining absorbing aerosols

Qirui Zhong *et al.*

Corresponding author: Qirui Zhong, q.zhong@vu.nl

Sci. Adv. **9**, eadi3568 (2023)
DOI: 10.1126/sciadv.adi3568

This PDF file includes:

Supplementary Text S1 and S2
Figs. S1 to S25
Tables S1 to S4
References

Supplementary Text

Supplementary Text 1. SSA errors and impacts of selected factors

As the modeled MAC is closely related to SSA, we investigate here the SSA errors in the AeroCom models. In **Fig. S6**, the modeled relationship between SSA and rBC is compared with in situ observations by ref (*24*), with most models showing underestimated SSA for a given rBC. The underestimation of SSA in the models becomes even more significant with increasing rBC. The highly absorbing aerosols in Africa therefore suffer more from such an error than those in the Amazon. This error is unlikely to be addressed by adjusting the rBC in emissions, as done by previous studies (*62*).

Essentially, the relationship between SSA and rBC can be affected by several factors. In this study, we aim to investigate the impacts of three key factors, namely particle size distribution, complex refractive index for BC, and mixing state, on this relationship. To accomplish this, we employ a series of Mie calculations using the Mätzler (*63*) model to determine the cross section of aerosol extinction and absorption for mixtures of BC and OA with varying combinations of the aforementioned factors. We assume a spherical particle shape for all Mie calculations, as suggested by the low depolarization ratios from the Cloud Aerosol Lidar with Orthogonal Polarization (CALIOP) observations (*64*). The hygroscopic growth is considered using the Köhler theory (*65*), which is found to have little impact on the calculations. For mixing states, we consider three assumptions used in AeroCom models (see **Table S1**): 1) external mixing, which assumes BC and OA particles exist separately; 2) homogeneous internal mixing, which assumes BC and OA particles are mixed at a molecular level, and the volume-weighted averages are used to calculate the refractive index of the mixed particles; and 3) core-shell structure with BC as the core in the center and other components (e.g., OA) as the coated shell of the particles. In general, the latter two cases are both referred to as internal mixing.

As shown in **Fig. S2A2**, particle size distribution is a crucial determinant of the relationship between SSA and rBC, where smaller particles tend to exhibit greater absorption. This partly accounts for the SSA underestimation in most models, as they tend to produce particles that are too small (*31*). In addition, the BC refractive index can directly affect SSA (**Fig. S2B2**). These two factors combined can generally explain the opposite SSA errors in the ECHAM-HAM and SPRINTARS models, with the former producing small particle sizes (high AE) and high refractive index, and the latter featuring large sizes (the lowest AE in the AeroCom ensemble) and the lowest refractive index. Regarding the impacts of mixing state, both the homogeneous internal mixing and core-shell structure produce lower SSAs than external mixing. Such a difference stands out particularly with a larger particle and higher refractive index. Although the external mixing shows better agreement (higher SSA) with the observations, in situ and laboratory measurements have frequently confirmed the internal-mixing structure for BBA (*66-70*). In addition, models assuming an external mixing do not show superior performance compared with other models. For the two internal mixing states, small differences are found in the predicted SSA with a low BC fraction, as reported by a previous study (*71*). Within the range of refractive indices used by the AeroCom models (**Table S1**), both internal mixing states still fail to reproduce the observed SSA. We further reduce the imaginary part of the BC refractive index to 0.3, as suggested by field measurements (*58,59*), which reduces the SSA error. The impacts of different refractive indices are also tested in ECHAM-HAM via sensitivity tests (**Fig. S23**), where the best agreement between the model and

observations is found when a refractive index of $0.3i$ is used. Based on the analysis, we correct the particle size and use the new refractive index in the two global models (ECHAM-HAM and SPRINTARS) to improve the modeled SSA (and MAC).

Supplementary Text 2. Predicting AAOD in African outflow for AeroCom models

Following our previous study, we adopt a meta-model analysis to predict the African outflow AAOD for the AeroCom models (*31*). A linear regression is established for the outflow AAOD ($AAOD_O$) as a function of emission (E_S), lifetime (τ_S), and MAC (MAC_S) in the source region using AeroCom model data (**Fig. S7A**). The general form of the regression can be written as:

$$AAOD_o = a \times E_s \tau_s MAC_s + b \times E_s \tau_s + c \times MAC_s + d$$

where *a*, *b*, *c*, *d* are the coefficients obtained from the regression using all model data. The detailed formula derivation can be found in ref (*31*). Then, the constrained values for E_S, τ_S, and MAC_S are used to predict the outflow AAOD. In **Fig. S7B**, we validate the predicted AAOD and the original AeroCom model output against satellite observations and an improved agreement is found for the prediction based on our constrained results. This directly demonstrates the utility of our method to predict the AAOD outflow. In addition, it verifies the reliability of the three constrained components (E_S, τ_S, and MAC_S) over the source region.

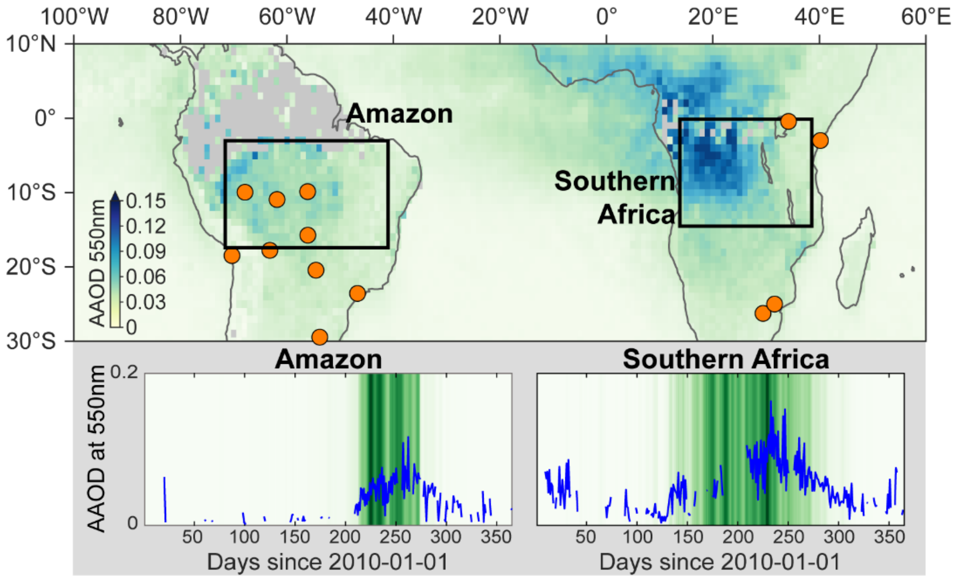

Fig. S1 Geographical distribution of annual mean AAOD from POLDER-GRASP observations (top) and daily series of AAOD over the two focused fire regions (bottom). The orange dots in the top map show the locations of AERONET monitoring sites considered for validating satellite datasets. The shaded scale in the background of the bottom diagrams reveals the normalized emission intensity based on GFED (with dark color indicating a high emission).

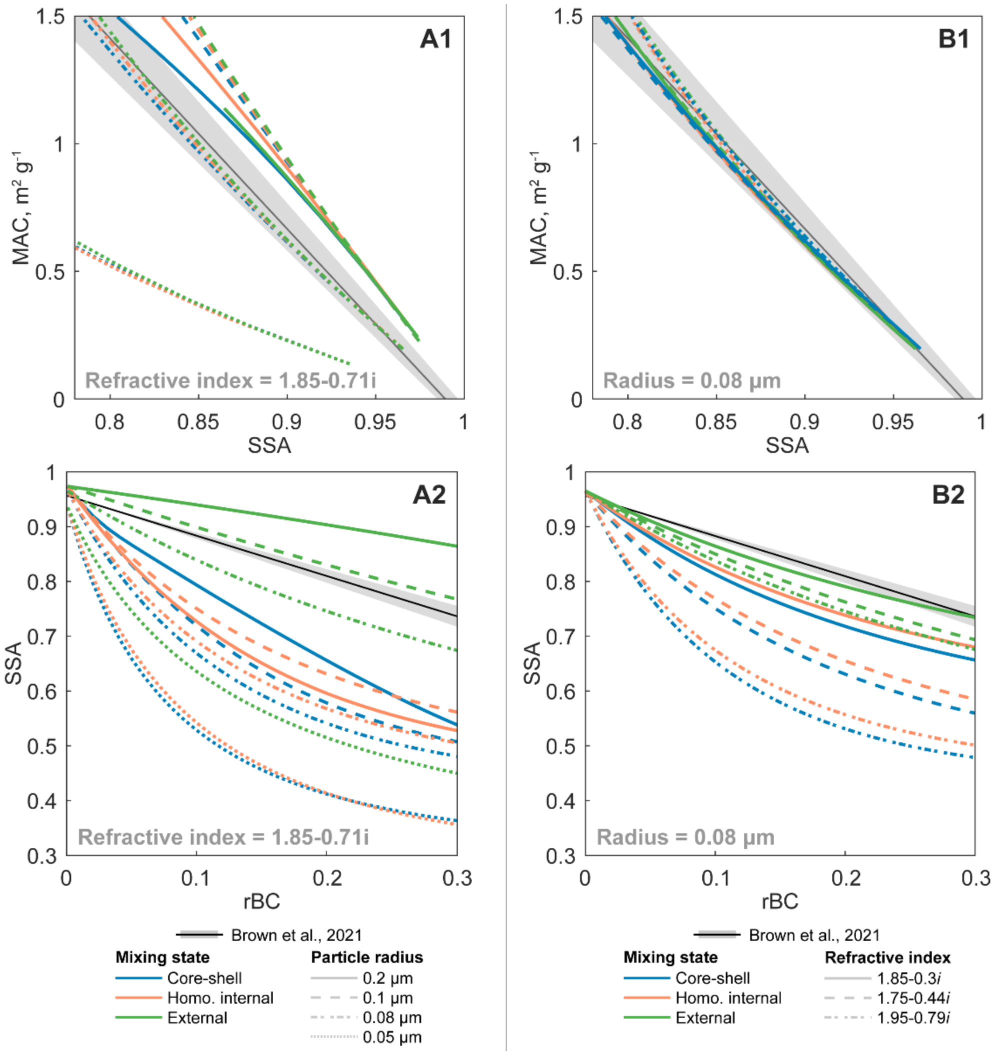

Fig. S2 Relationships between MAC and SSA (A1, B1) and between SSA and rBC (A2, B2) affected by mixing state and particle size (A1, A2) and by mixing state and complex refractive index (B1, B2). The rBC is calculated as BC:[BC+OA]. All the relationships are calculated at a wavelength of 550 nm based on the idealized Mie model for three mixing states: core-shell structure, homogeneous internal mixing, and external mixing (see Materials and Methods). Observations are taken from ref (*24*). Note that the observations by ref (*24*) consider background aerosols (< 15%), while our calculations are only for the mixture of BC and OA. In Fig. A1-A2, we assume a refractive index of 1.85-0.71*i,* which is also used in the ECHAM-HAM model and represents moderate absorption in the model ensemble. In Fig. B1-B2, we assume a radius of 0.08 µm, as suggested by ref (*24*). The three refractive indices in Fig. B1-B2 indicate the most absorbing (1.95-0.79*i*) and least absorbing (1.75-0.44*i*) in AeroCom models, and a suggested value based on in situ studies (1.85-0.30*i*). There is an extreme case in Fig. A1 using the 0.05 µm radius, which is typically smaller than the reported BBA size.

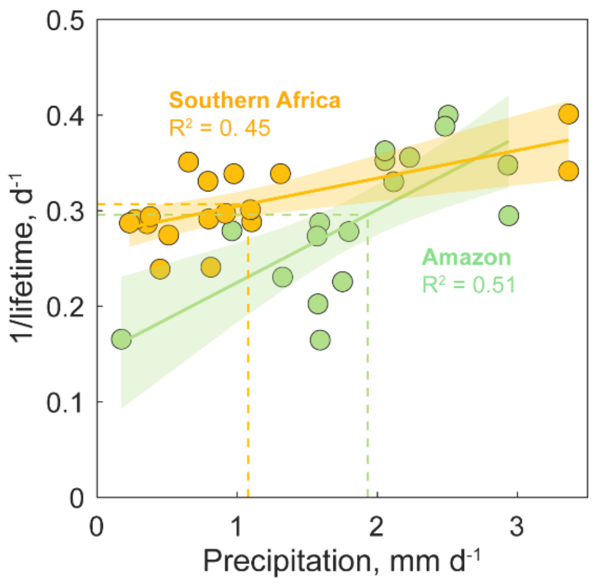

Fig. S3 Relationships between modeled lifetime and modeled precipitation in the Amazon and Southern Africa. Each dot represents the seasonally averaged data from a single model, with colors indicating the two fire regions. Lifetime is calculated as total burden divided by total emissions for BC and OA only. Solid lines are the regressions built between 1/lifetime and precipitation together with 95% confidence intervals (shaded area). Vertical dashed lines denote the GPCP observations for precipitation, and the horizontal dashed lines show the constrained 1/lifetime values. The R-squares (R^2) of the regressions are shown for the two regions. Note that real lifetime regression uses both precipitation and the Angstrom Exponent (see Materials and Methods).

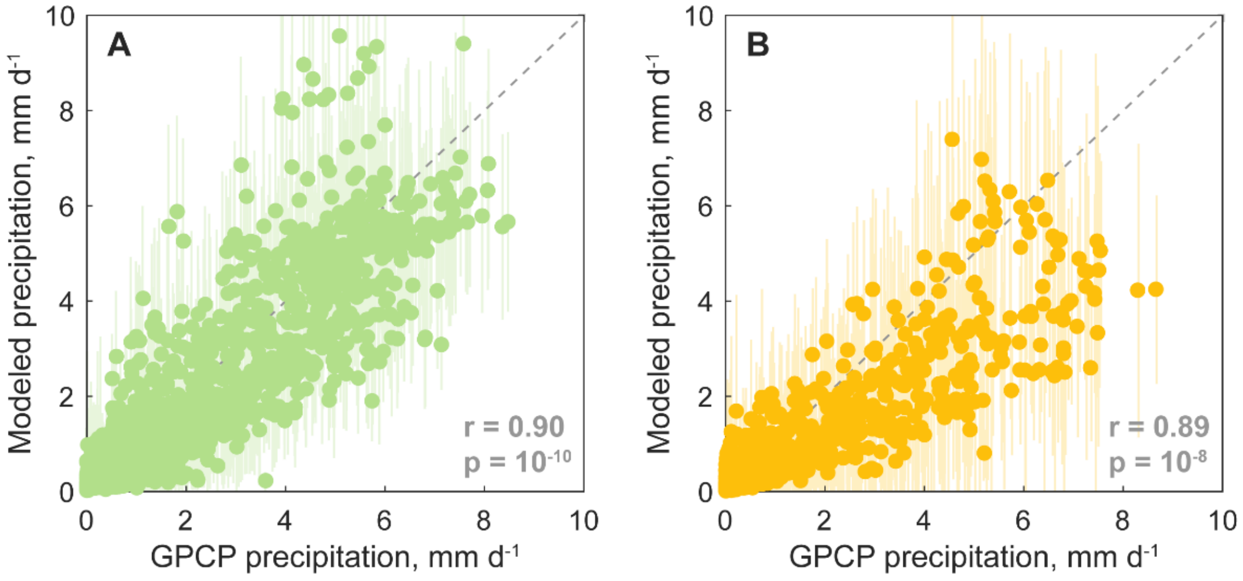

Fig. S4 Comparisons of modeled precipitation with observations from GPCP for the Amazon (A) and Southern Africa (B). Each dot represents the monthly average precipitation over a 1°×1° grid box from either the GPCP dataset or the multi-model average from 17 AeroCom models. The vertical bars show the corresponding standard deviations of the 17 individual models. Dashed lines indicate the 1:1 ratio. The correlation coefficients (r) and p value (p) are shown for the two regions. Data are only considered for fire seasons.

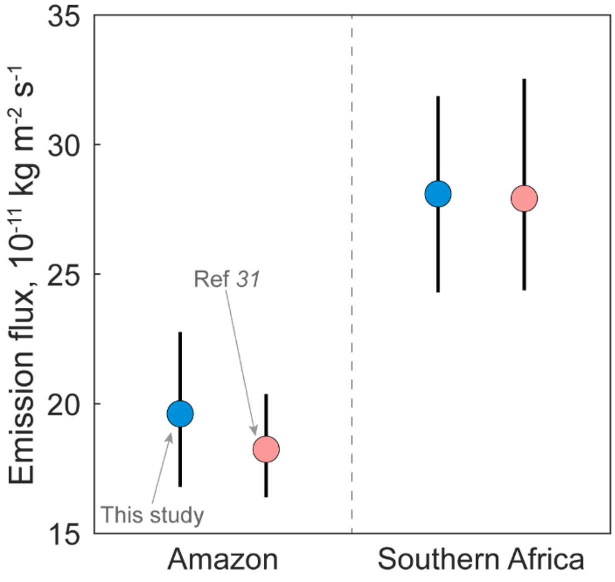

Fig. S5 Comparisons of constrained emissions between this study and our previous work over the Amazon and Southern Africa. The results from our previous work (*31*) are based on AOD and total aerosol extinction, which differs from this study. The error bars indicate the interquartile ranges considering all the uncertainty factors. Note that the emissions from ref (*31*) represent all aerosol species, while the results from this study are for BC+OA only (the latter is the dominant component of the former).

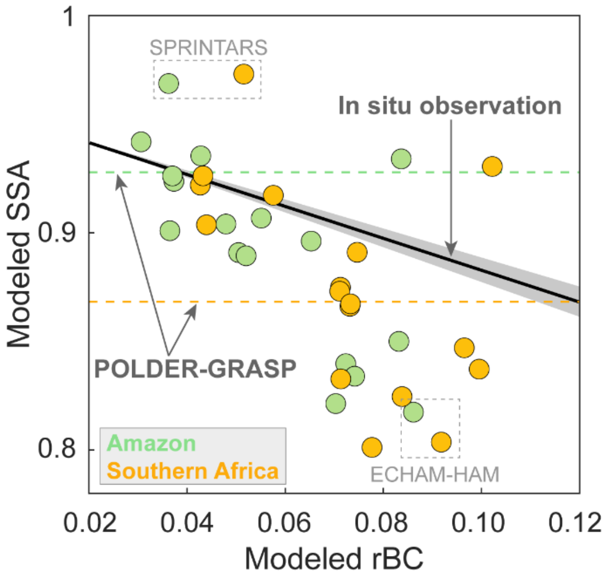

Fig. S6 Comparison of modeled relationship between SSA and rBC with observed relationship. The rBC is calculated as BC:[BC+OA]. Each data point represents the data averaged over the fire season, with the dot color indicating the two BB regions (green for Amazon and orange for Africa). The observed relationship from ref (*24*) is shown as a solid line with the 95% confidence interval (gray shaded area). SSA observations from POLDER-GRASP are shown as horizontal dashed lines. The ECHAM-HAM and SPRINTARS produce the most negative and positive SSA errors in the AeroCom model ensemble and are further corrected in this study.

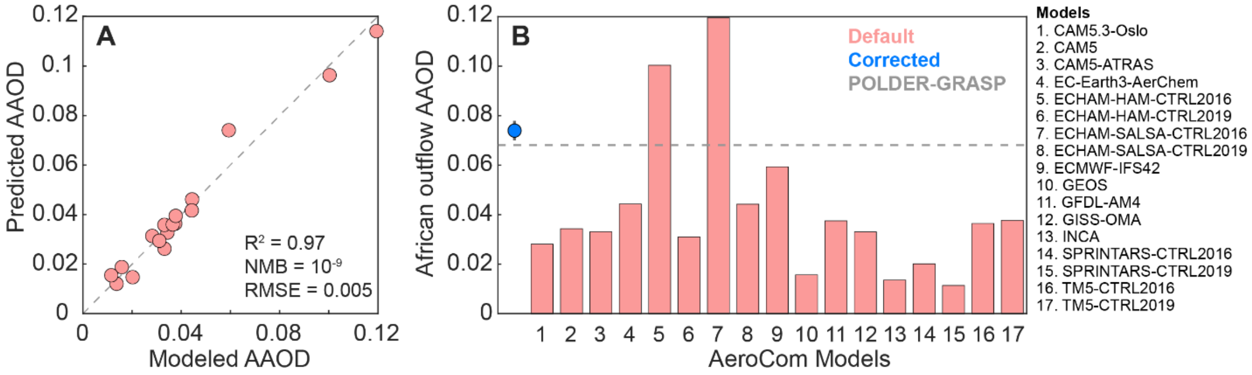

Fig. S7 Linear regression for the African outflow AAOD based on AeroCom models (A) and the predicted outflow AAOD using the regression (B). In Fig. A, the regression has the form of $AAOD_o = A\times E_s\tau_s\ MAC_s+B\times E_s\tau_s+C\times MAC_s+D$, where $AAOD_o$ indicates the outflow AAOD; E_s, τ_s, and MAC_s are the total emission, lifetime, and MAC over the source region; and A, B, C, and D are the regression coefficients (see Supplementary Text 2). The metrics show the R-square (R^2), normalized mean bias (NMB), and root mean square error (RMSE) for the regression. In Fig. B, constrained emission, lifetime, and MAC over the source region are adopted to the regression to predict the AAOD in the outflow area as a corrected case (blue dot), with the error bar showing the interquartile range of the prediction. The predicted AAOD value is compared with the default model data (red bars) and satellite observation (gray dashed line).

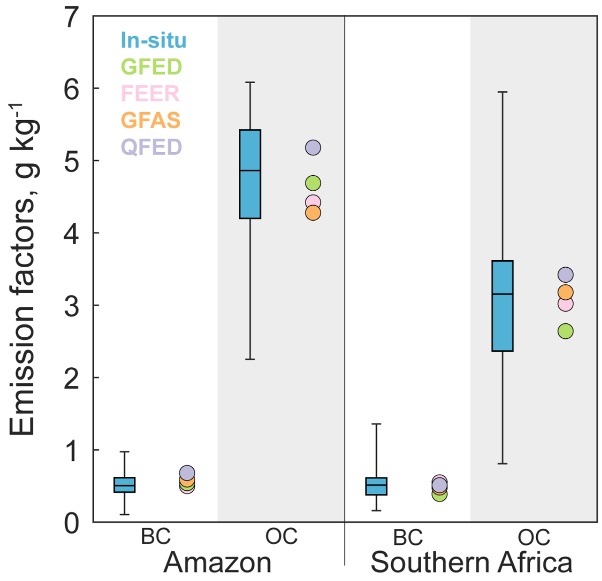

Fig. S8 Comparisons of emission factors between emission inventories and in situ measurements over the Amazon and Southern Africa. The in situ results are shown as box plots. The box plots show the 5-95% (whiskers), 25-75% percentile ranges (solid rectangles) and the median values (solid horizontal lines) based on the data collected from previous studies (Table S2). The data measured for tropical forest/deforestation fires and savanna/grassland fires are used in the analysis over the Amazon and Africa, respectively (Table S2).

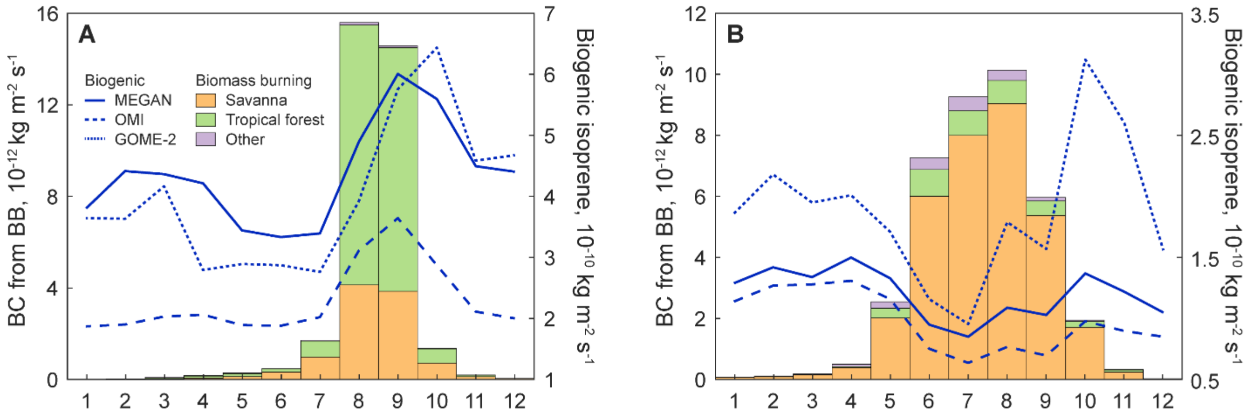

Fig. S9 Monthly evolution of biomass burning BC emissions and biogenic isoprene emissions over the Amazon (A) and Southern Africa (B). The emissions are shown as monthly flux averaged for each region. Biomass burning emissions are based on GFED4.1s (http://www.globalfiredata.org/), which are further divided into three fire types (savanna, tropical forest, and other). Biogenic emissions are obtained from one bottom-up estimate based on MEGAN model driven by ERA5 and two top-down estimates using constraints from OMI and GOME-2 satellite data (https://emissions.aeronomie.be/).

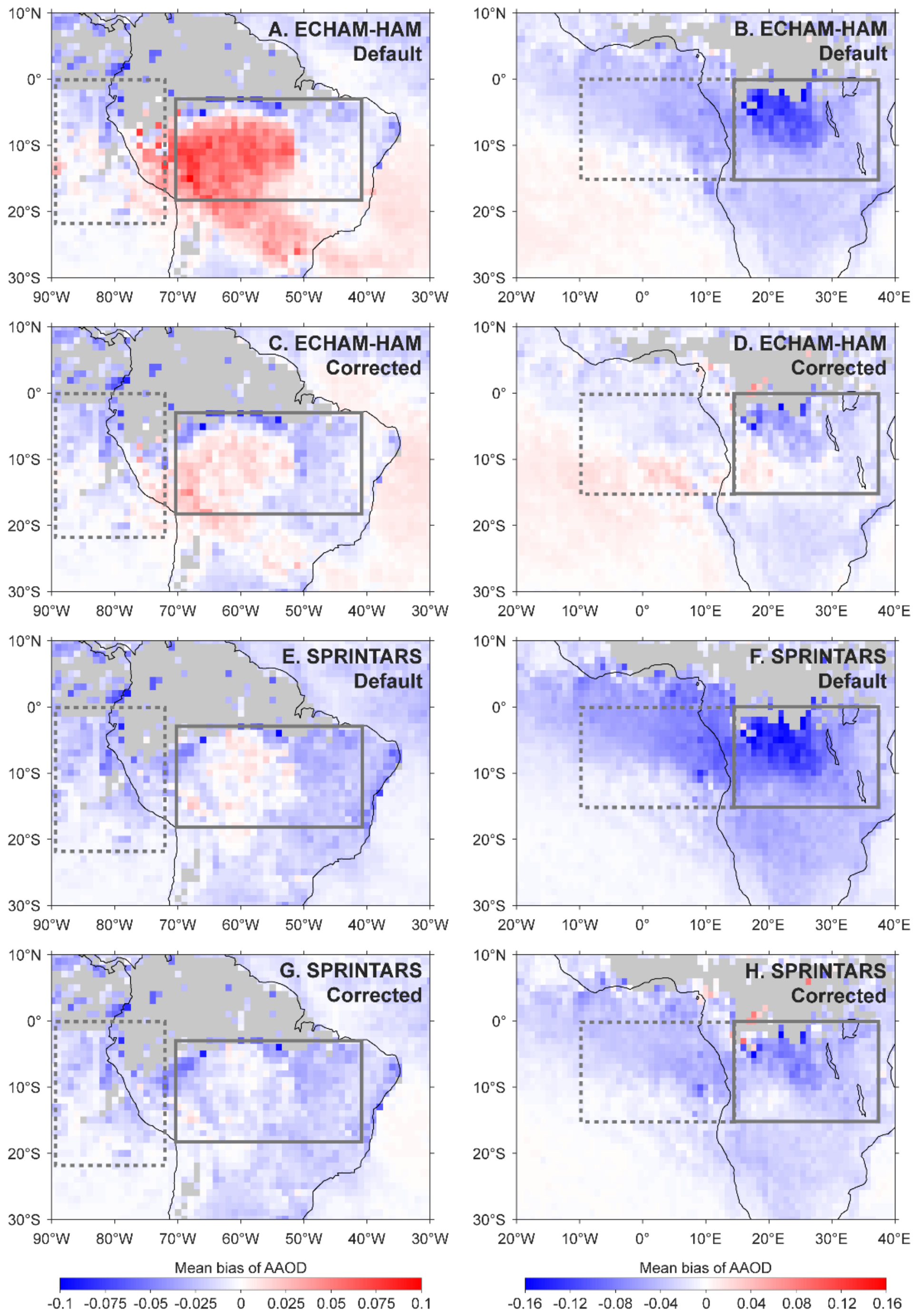

Fig. S10 Seasonal mean bias for AAOD over the Amazon (A, C, E, G) and Africa (B, D, F, H) from ECHAM-HAM (A-D) and SPRINTARS (E-H). The results for both default and corrected cases are shown. The data are collocated with POLDER-GRASP at 1°×1°×daily resolution. Gray areas indicate regions with no available observations. The gray boxes with solid edges show the source regions where we conduct the error analysis. The boxes with dashed edges show the corresponding outflow regions of focus in this study.

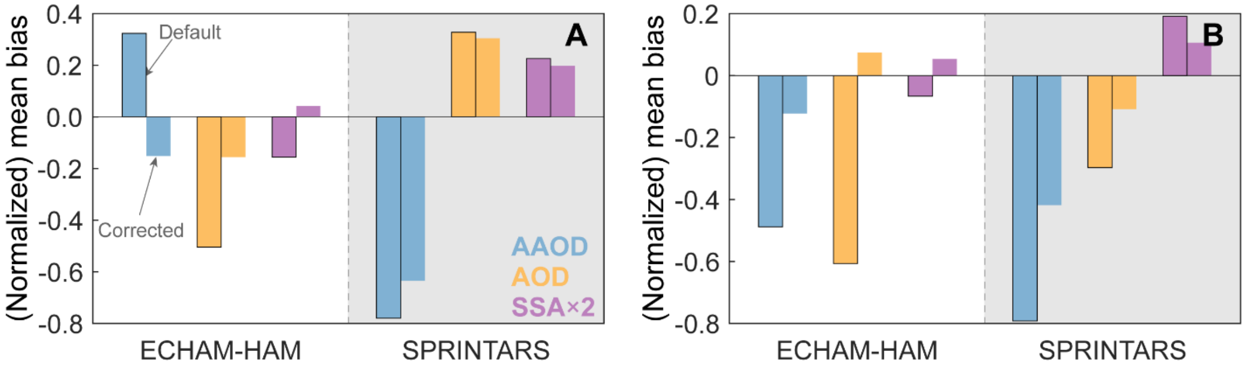

Fig. S11 Seasonal mean bias of AAOD, AOD, and SSA for default and corrected simulations by ECHAM-HAM and SPRINTARS over the outflow regions of the Amazon (A) and Southern Africa (B). The results are shown in the same format as Fig. 5 but for outflow regions as indicated in Fig. S10. Model data are collocated with the POLDER-GRASP dataset during the fire seasons.

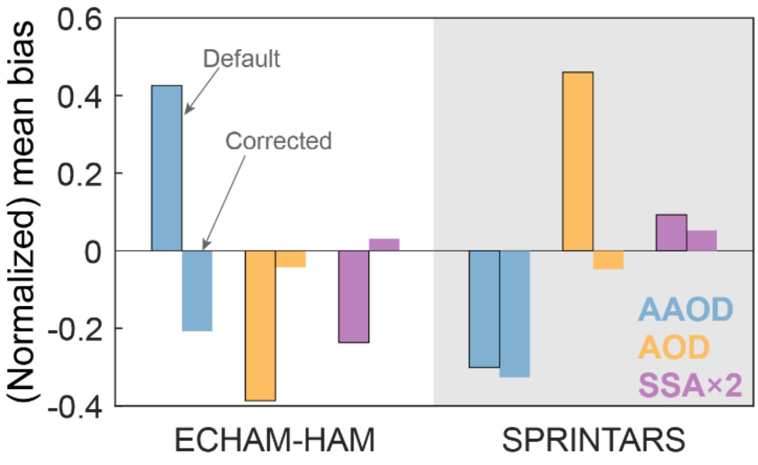

Fig. S12 Seasonal mean bias of AAOD, AOD, and SSA for the default and corrected ECHAM-HAM and SPRINTARS simulations compared with AERONET data. The results are shown in the same format as Fig. 5A, except for that the observations are taken from AERONET sites as shown in Fig. S1.

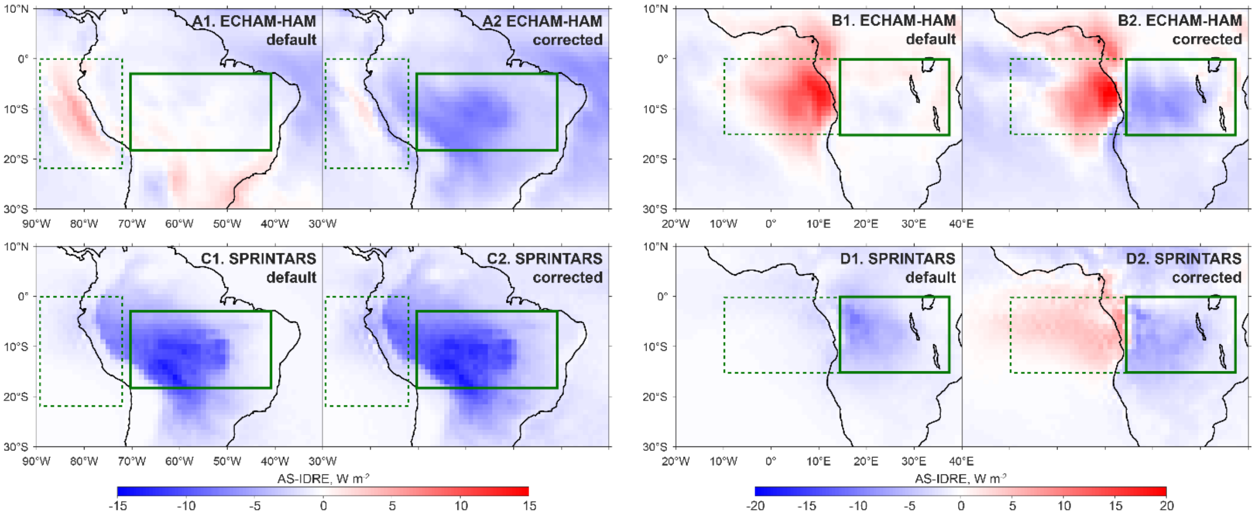

Fig. S13 Seasonal mean IDRE over the Amazon (A, C) and Africa (B, D) from the ECHAM-HAM and SPRINTARS models for the default and corrected cases. The all-sky IDREs (AS-IDRE) are shown as averages over the fire seasons. The green boxes show the source regions. The boxes with dashed edges indicate the corresponding outflow regions of focus in this study.

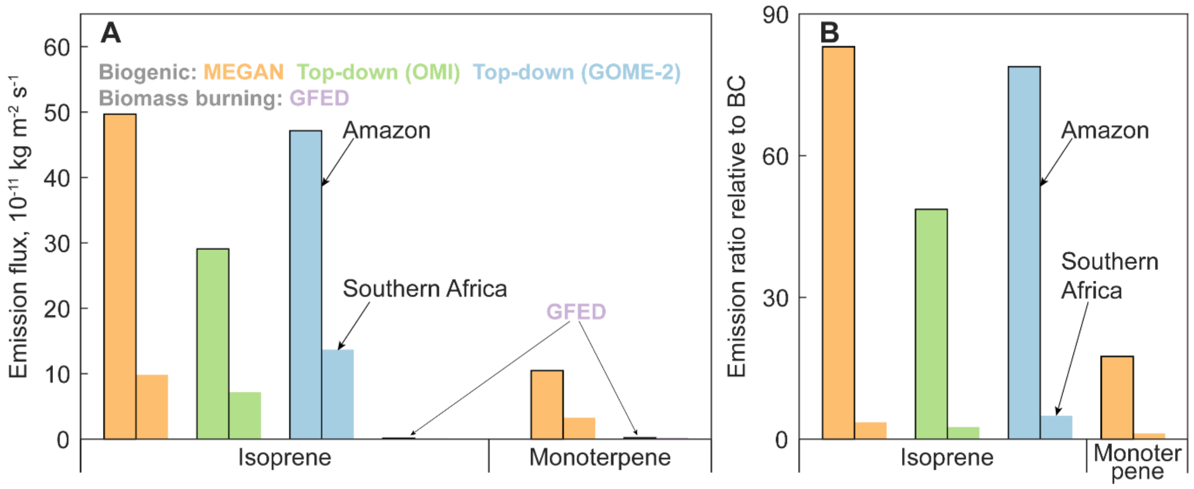

Fig. S14 The emissions for precursor gas relevant to SOA formation. A) The emissions of isoprene and monoterpene are shown for the Amazon (bar with a solid edge) and Southern Africa (bar without edge) for both biogenic and biomass sources. For biogenic emissions, one bottom-up (MEGAN) and two top-down (constrained by OMI and GOME-2 satellite observations, respectively) datasets are presented (*72-74*). For comparison, the GFED BB emissions are shown (which are much lower than biogenic emissions). All emissions are shown as the regional average flux over the fire seasons. B) The ratio of the biogenic isoprene and monoterpene emissions to constrained BC emissions. The sources of biogenic emissions are the same as Fig. A.

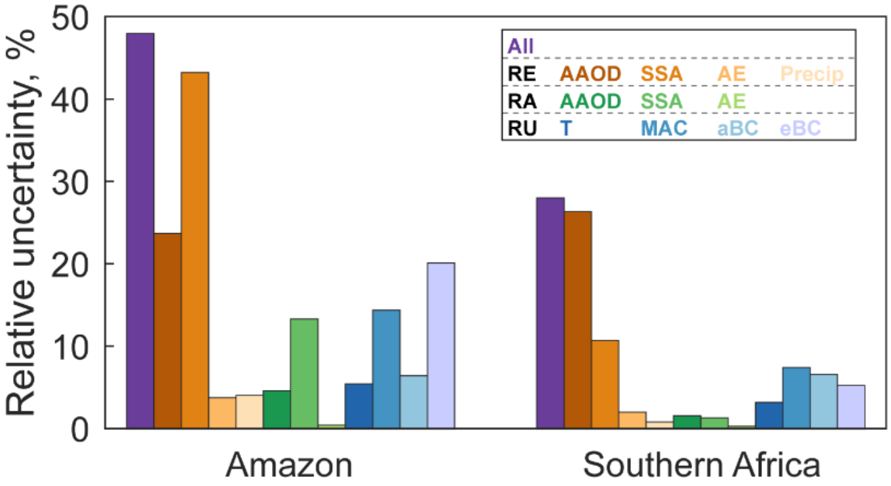

Fig. S15 The relative uncertainties of constrained BC emissions due to all and individual uncertainty sources. The uncertainties are shown as interquartile divided by median. The overall uncertainties are shown as purple bars (All). Individual uncertainty factors are considered for retrieval error (RE) of AAOD, SSA, AE, and precipitation; uncertainties of the regional averages (RA) for AAOD, SSA, and AE; the regression uncertainties (RU) for constrained lifetime (T), MAC, ambient BC:[BC+OA] (aBC), and emitted BC:[BC+OA] (eBC).

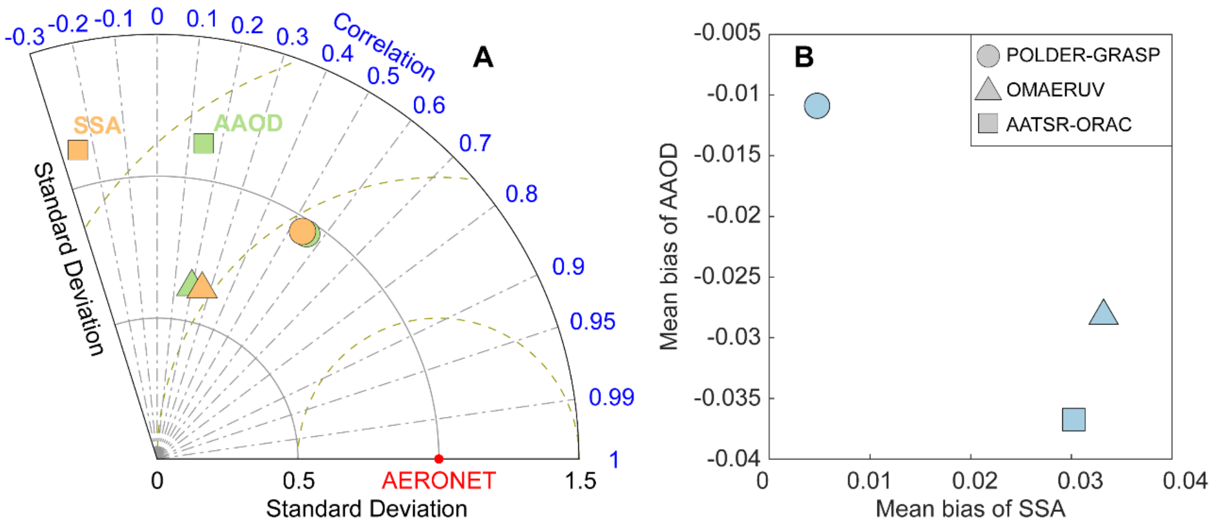

Fig. S16 Validation of three satellite products against AERONET dataset. The validation is shown as a Taylor diagram for AAOD (light green) and SSA (orange) in Fig. A and a scatter plot for mean bias in Fig. B. The shape of the symbols indicates different satellite products. All three satellite products and the AERONET dataset are collocated with each other during the fire season to ensure the same sampling.

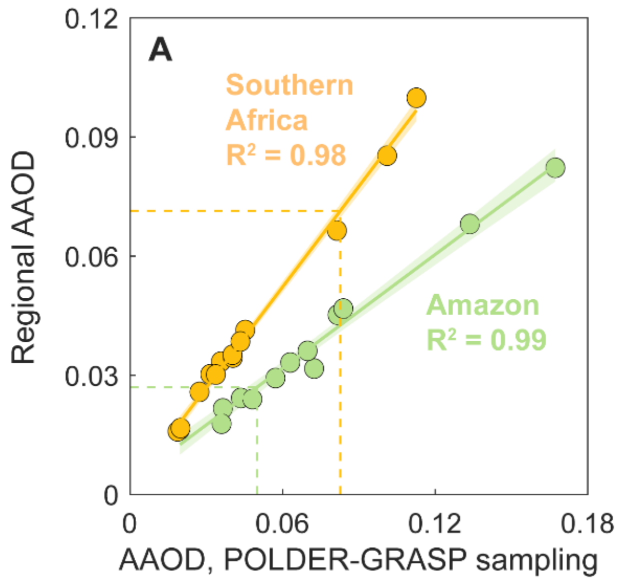

Fig. S17 Relationship between averages of regional AAOD (A) and SSA (B) and POLDER-GRASP sampled values in AeroCom. Each dot represents the average value from an individual model. For the sampled AAOD and SSA, model data during fire seasons are collocated with POLDER-GRASP on a daily basis. The solid lines show the linear regressions with 95% confidence intervals (shaded areas). The R-squares (R^2) of the regressions are shown individually for the two fire regions. Vertical dashed lines show the average values of the raw POLDER-GRASP data, and the horizontal dashed lines indicate the predicted regional values.

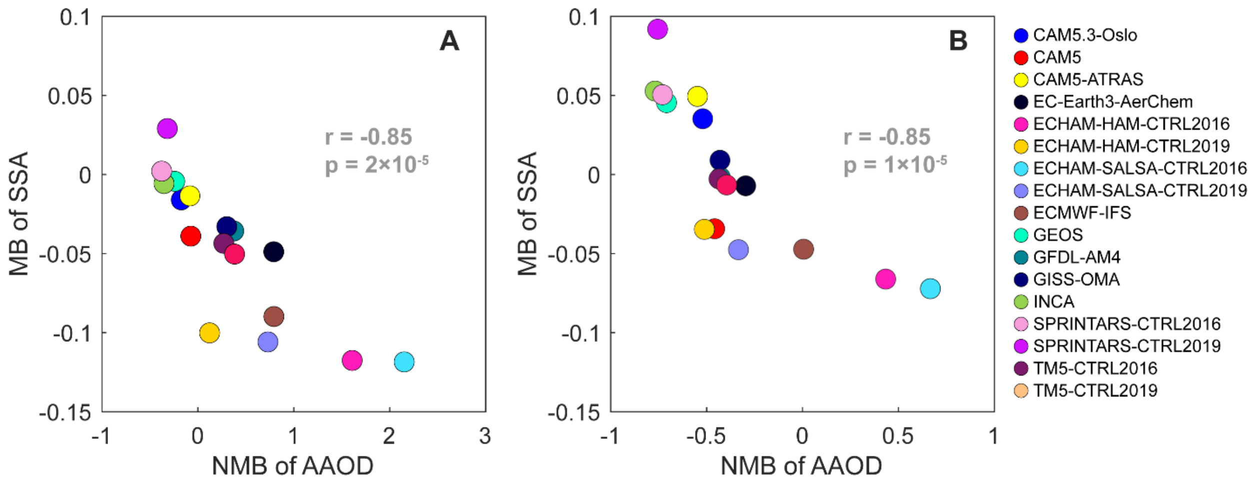

Fig. S18 Modeled mean bias (MB) of SSA and normalized mean bias (NMB) of AAOD over the Amazon (A) and Southern Africa (B) during fire seasons. Biases are calculated based on the non-collocated model data and reconstructed regional observations using POLDER-GRASP retrievals (see Fig. S17).

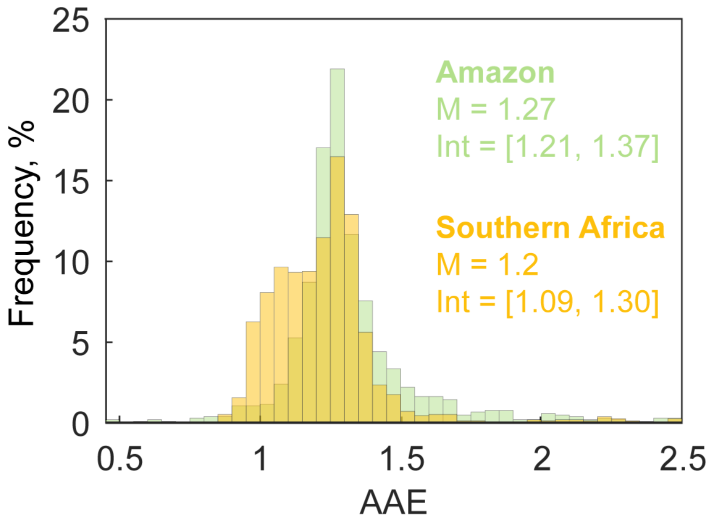

Fig. S19 Distribution of the absorption angstrom exponent (AAE) from POLDER-GRASP over the Amazon and Southern Africa. The AAE is calculated using the AAOD at 440/870 nm wavelength based on the daily data from individual grid cells during fire seasons. Statistics show the median (M) and interquartile ranges (Int) of the distributions.

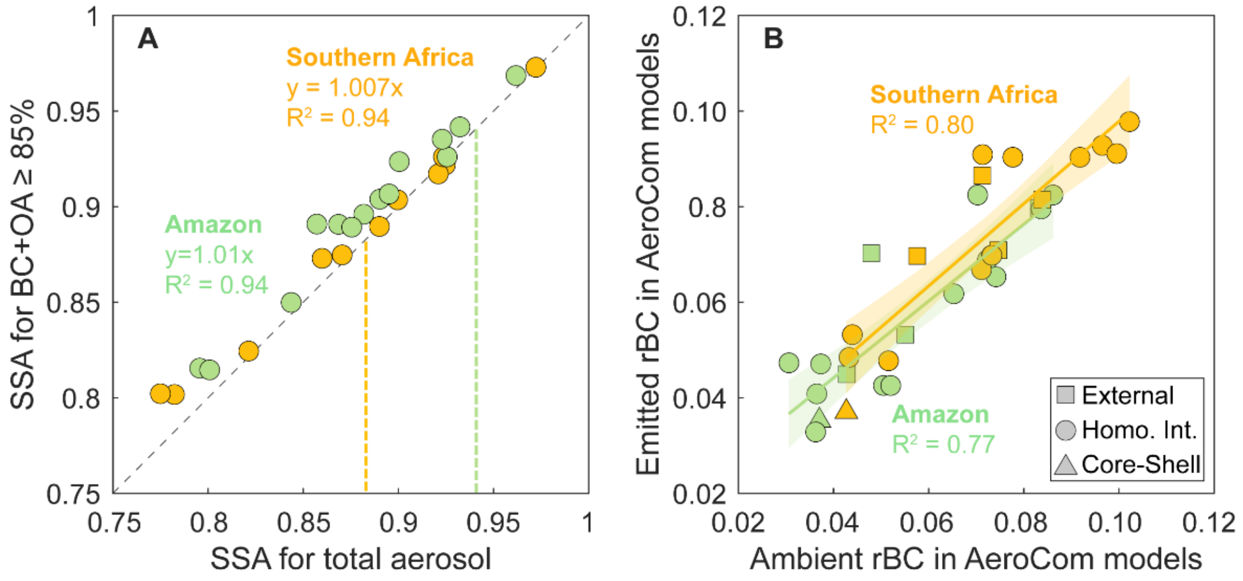

Fig. S20 Relationships between SSA in all grid cells and SSA in grid cells with BC + OA ≥ 85% total aerosols (A) and the dependence of ambient rBC on emitted rBC (B). The rBC is calculated as BC:[BC+OA]. The vertical dashed lines in Fig. A show the regional SSA observations from POLDER-GRASP. The black dashed line indicates the 1:1 line. In Fig. B, the linear regressions between emitted and ambient rBC are shown as solid lines with 95% confidence intervals (shaded areas).

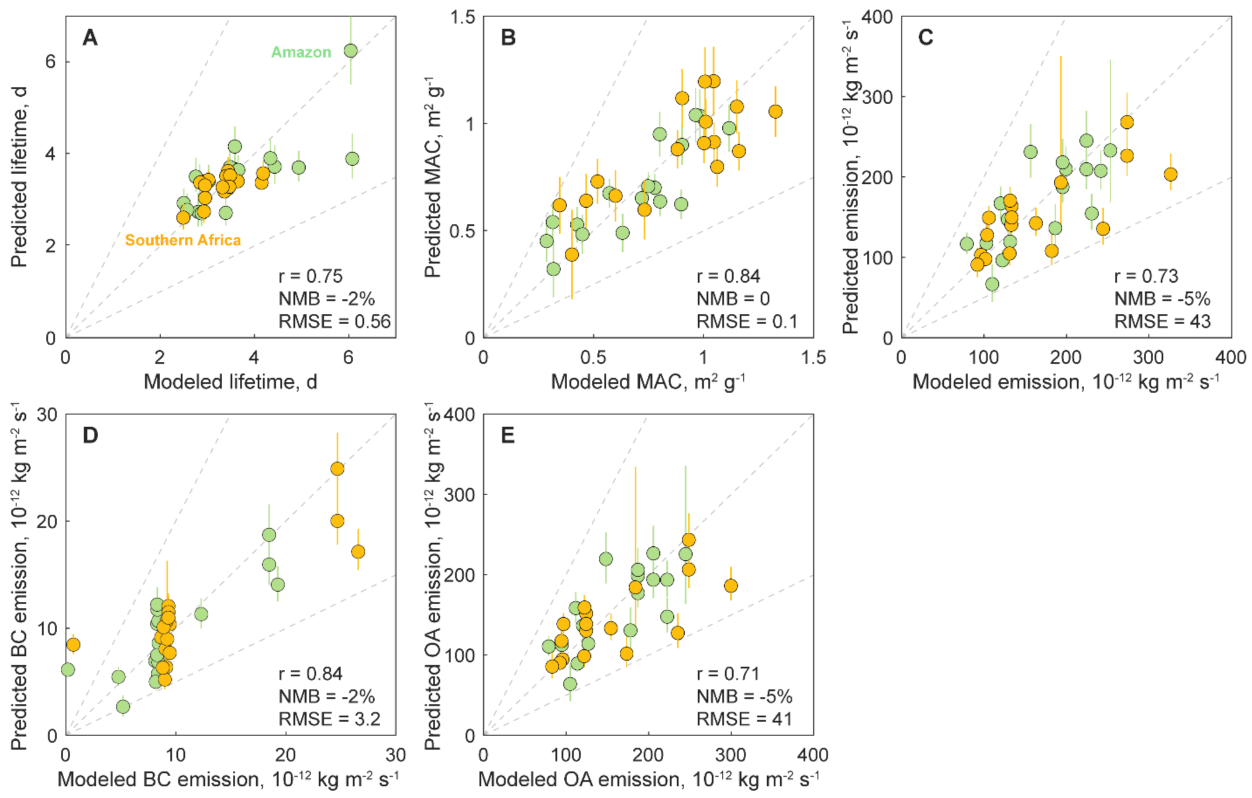

Fig. S21 Comparisons of predicted lifetime (A), MAC (B), total emission (C), BC emission (D), and OA emission (E) using the constraining procedures with the original model data. The predicted values for each model are estimated with all the other models following the constraining procedure. Vertical error bars denote the 95% prediction intervals. The dashed lines indicate the 1:1, 1:2, and 2:1 range. Metrics include the Pearson correlation coefficient (r), normalized mean bias (NMB), and root mean square error (RMSE).

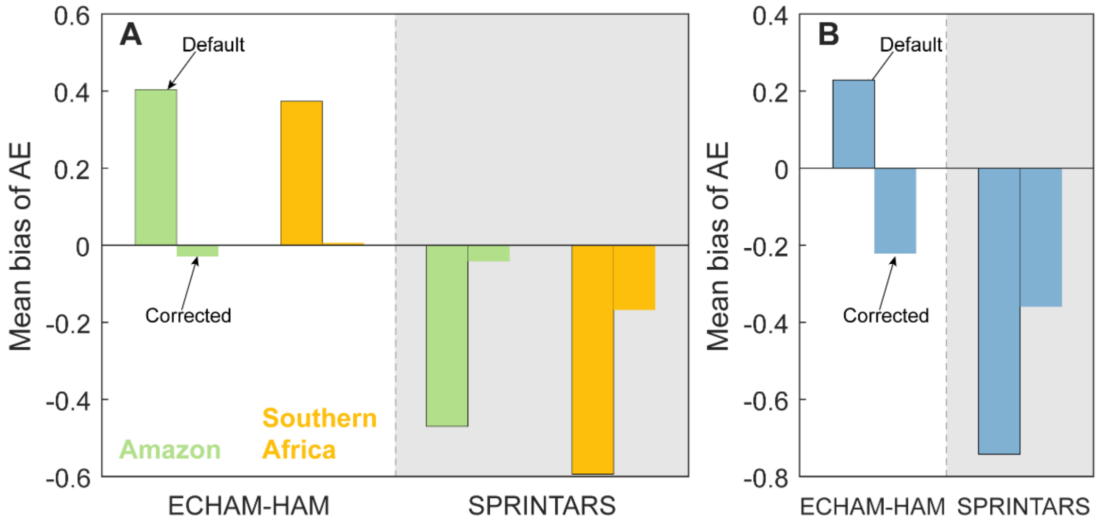

Fig. S22 Seasonal mean bias for the Angstrom Exponent (AE) in ECHAM-HAM and SPRINTARS for default (bars with solid edges) and corrected simulations (bars without edges). The model data are collocated and compared with POLDER-GRASP (A) and AERONET (B). Angstrom Exponent is calculated based on AOD at 440 and 550 nm wavelengths. The detailed configurations of the ambient particle size for the default and corrected cases can be found in Table S3. The comparison with POLDER-GRASP is conducted separately for the two regions as indicated by bar color. The AERONET observations are collected from the sites as shown in Fig. S1, which are mostly in the Amazon area.

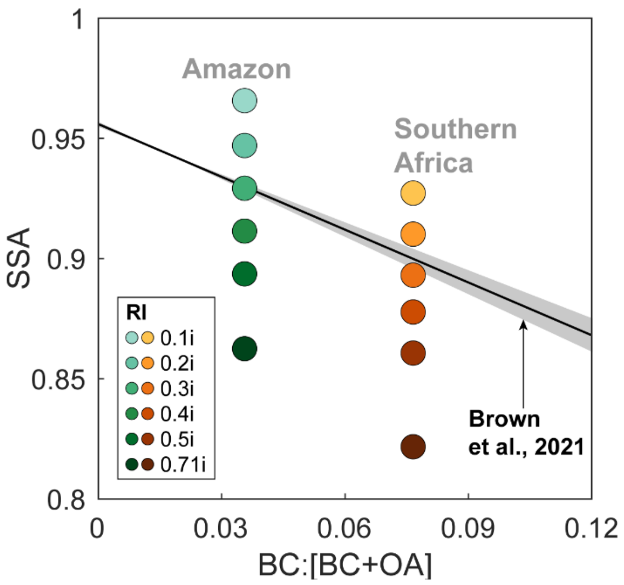

Fig. S23 Simulated changes in SSA in response to the different imaginary parts of the BC refractive index (RI) in the ECHAM-HAM model. The colors of the circles denote data over two fire regions with color scales indicating different refractive indices. To highlight the impacts of the refractive index, the SSA is simulated using constrained emissions, modified particle size, and rescaled precipitation (Materials and Methods). In particular, the particle size is modified based on the modeled AE. The choice of refractive index of BC can affect the modeled AE, but the impact is generally small and would not fundamentally alter our results.

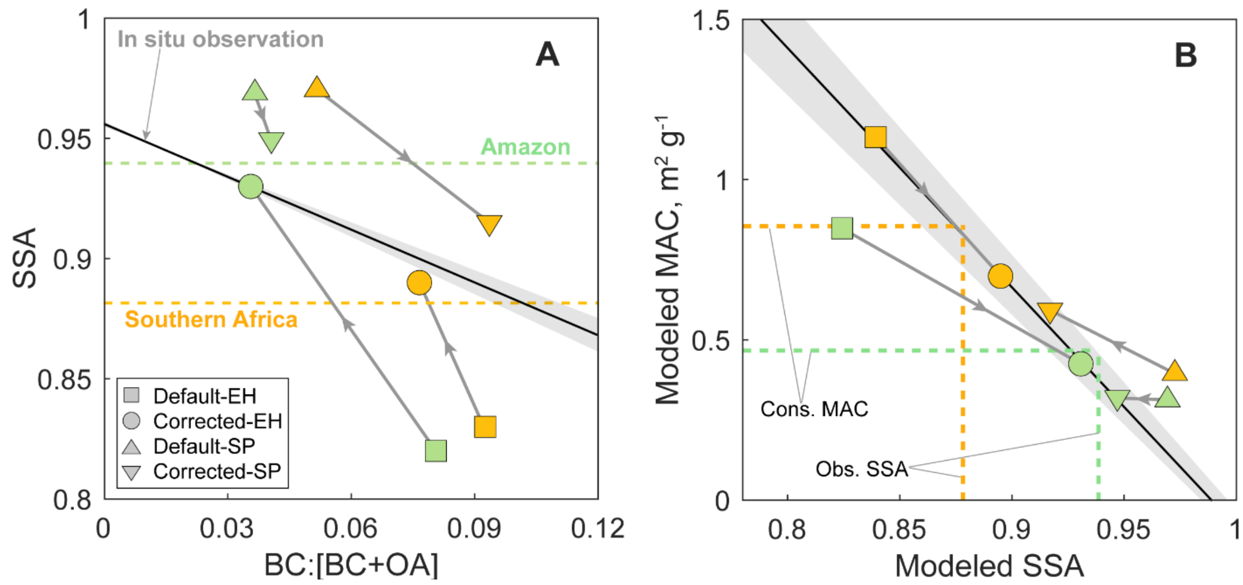

Fig. S24 Modeled relationships between SSA and BC:[BC+OA] (A) and between MAC and SSA (B) using default and corrected configurations for ECHAM-HAM (EH) and SPRINTARS (SP). The results are shown for the direct model output without collocations. For the corrected cases, models are corrected with rescaled particle size distribution, refractive index, modified precipitation, and constrained BC and OA emissions (see Materials and Methods). The black solid lines indicate the observations from ref (*24*) with 95% confidence intervals (gray shaded areas). Data for the two fire regions are shown in different colors. Regional observations of SSA based on POLDER-GRASP are shown as horizontal dashed lines in Fig. A and vertical dashed lines In Fig. B. The constrained MACs are shown as horizontal dashed lines in Fig. B. Note that the constrained MAC is predicted based on model relationship instead of the observed relationship as shown. The arrows in both plots indicate the direction from default to corrected simulations.

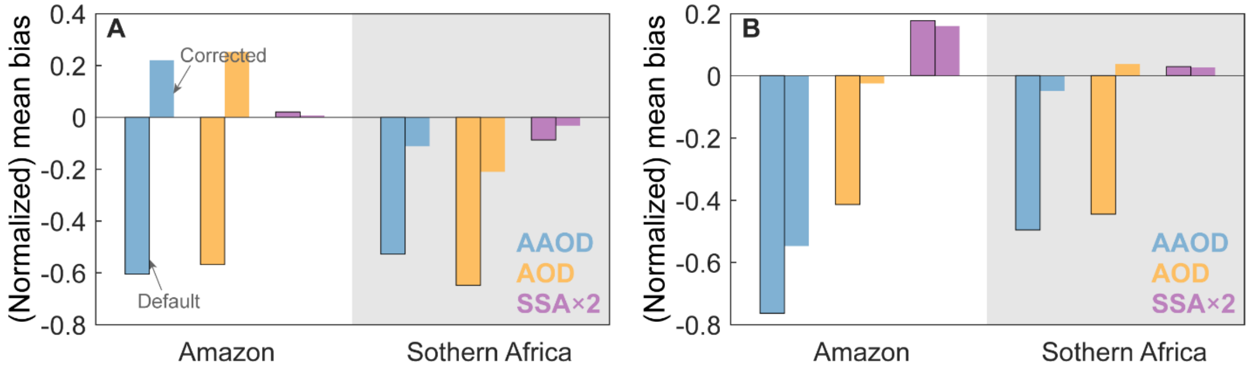

Fig. S25 Seasonal mean bias of AAOD, AOD, and SSA from the default and corrected ECHAM-HAM simulations over the source (A) and outflow regions (B) of the Amazon and Southern Africa. Model data are collocated and compared with POLDER-GRASP observation for 2009 fire season. The source and outflow regions are shown in Fig. S10.

Table S1. Information on biomass burning aerosols in the 17 AeroCom models used in this study. The experiment name (CTRL2016, CTRL2019) is added to the names of the models if they participate in both experiments. Note that the information is for biomass burning aerosols only.

Models	Mixing state for BC	Emission	OA/OC	Refractive index	Ref
**CAM5.3-Oslo**	Internal mixing with Maxwell-Garnett rule	CMIP5	2.6	OA: 1.53-0.006*i*<br>BC: 1.95-0.79*i*	*75*
**CAM5**	Internal mixing	CMIP6	1.4	OA: 1.53-0.005665*i*<br>BC: 1.95-0.79*i*	*76*
**CAM5-ATRAS**	Core-shell for internally-mixed BC particles	CMIP6	1.4	OA: 1.53-0.005665*i*<br>BC: 1.95-0.79*i*	*77*
**EC-Earth3-AerChem**	Internal mixing with Maxwell-Garnett rule	CMIP6	1.6	OA: 1.53-0.00554*i*<br>BC: 1.85-0.71*i*	*78*
**ECHAM-HAM-CTRL2016**	Internal mixing with volume-weighted average	3.4×GFAS	1.4	OA: 1.53-0.0055*i*<br>BC: 1.85-0.71*i*	*54*
**ECHAM-HAM-CTRL2019**	Internal mixing with volume-weighted average	CMIP6	1.4	OA: 1.53-0.0055*i*<br>BC: 1.85-0.71*i*	*54*
**ECHAM-SALSA-CTRL2016**	Internal mixing for each mode with volume-weighted average	3.4×GFAS	1.4	OA: 1.53-0.0055*i*<br>BC: 1.85-0.71*i*	*79*
**ECHAM-SALSA-CTRL2019**	Internal mixing for each mode with volume-weighted average	CMIP6	1.4	OA: 1.53-0.0055*i*<br>BC: 1.85-0.71*i*	*79*
**ECMWF-IFS**	External mixing	3.4×GFAS	1.8	OA: 1.5346-0.015*i*<br>BC: 1.75-0.45*i*	*80*
**GEOS**	External mixing	CMIP6	1.8	OA: 1.53-0.005*i*<br>BC: 1.75-0.45*i*	*81*
**GFDL-AM4**	All aerosols are externally mixed except for sulfate and black carbon which are internally mixed by volume weighting of refractive indices	CMIP6	1.4	OA: 1.53-0.006*i*<br>BC: 1.75-0.44*i*	*82*
**GISS-OMA**	External mixing, with a BC absorption amplification factor of 1.5	CMIP6	1.4	OA: 1.527-0.014*i*<br>BC: 1.85-0.71*i*	*83*
**INCA**	Internal mixing for BC with Maxwell-Garnett to compute its refractive index	CMIP6	1.4	OA: 1.53-0.0055*i*<br>BC: 1.75-0.44*i*	*84,85*
**SPRINTARS-CTRL2016**	Internal mixing of BC and OA with volume-weighted average for effective refractive index	GFED3.1	2.6	OA: 1.53-0.006*i*<br>BC: 1.75-0.44*i*	*55*

**SPRINTARS-CTRL2019**	Internal mixing of BC and OA with volume-weighted average for effective refractive index	CMIP6	2.6	OA: 1.53-0.006*i*<br>BC: 1.75-0.44*i*	*55*
**TM5-CTRL2016**	Internal mixing with Maxwell-Garnett rule	CMIP6	1.6	OA: 1.53-0.00554*i*<br>BC: 1.85-0.71*i*	*78,86*
**TM5-CTRL2019**	Internal mixing with Maxwell-Garnett rule	CMIP6	1.6	OA: 1.53-0.00554*i*<br>BC: 1.85-0.71*i*	*78,86*

Table S2. Emission factors of BC, OC, and OC/BC ratio for biomass burning in previous studies. Data (unit: g/kg dry matter burned) are collected from in situ measurements or emission inventories for tropical forest/deforestation and savanna/grassland fires regarding the major biomes burned in the Amazon and Southern Africa, respectively. Values used in the four emission inventories are shown in italics for comparison. The mean values and standard errors (SE) for emission factors of BC and OC across in-situ studies are used to estimate the OC/BC ratio and its uncertainty (see Materials and Methods).

**Tropical forest/deforestation**				**Savanna/grassland**			
**Ref**	**BC**	**OC**	**OC/BC**	**Ref**	**BC**	**OC**	**OC/BC**
87	0.99	3.56	3.6	*98*	0.69	3.16	4.56
88	0.96	6.08	6.34	*88*	0.16	2.82	17.31
89	0.59	6.08	10.38	*99*	0.37	3.28	8.88
90	0.47			*99*	0.45		
91	0.66	5.2	7.88	*100*	0.26		
92	0.46			*101*	0.61		
93	0.19	1.53	7.96	*102*	0.59	1.61	2.73
94	0.52	4.71	9.06	*89*	0.52	3.53	6.82
95	0.02	5	263.16	*90*	0.78		
96	0.38			*103*	0.59	1.61	2.73
97	0.51	4.4	8.63	*104*	0.39	2.3	5.9
**Mean**	**0.52**	**4.57**	**8.76**	*105*	0.24	0.7	2.92
**SE**	**0.09**	**0.53**		*105*	0.47	3	6.38
GFED	*0.52*	*4.71*	*9.06*	*106*	0.5	2.84	5.69
FEER	*0.51*	*4.4*	*8.63*	*94*	0.61	5.79	9.49
GFAS	*0.57*	*4.3*	*7.54*	*107*	1.29	3.68	2.85
QFED	*0.66*	*5.2*	*7.88*	*108*	1.43	2.44	1.7
				109	0.16		
				110	0.53		
				111		3.56	
				95	0.13	0.82	6.31
				97	0.37	3.28	8.88
				**Mean**	**0.51**	**2.66**	**5.19**
				**SE**	**0.07**	**0.31**	
				GFED	*0.37*	*2.62*	*7.08*
				FEER	*0.53*	*3*	*5.66*
				GFAS	*0.46*	*3.2*	*6.96*
				QFED	*0.48*	*3.4*	*7.08*

Table S3. Model configurations for ECHAM-HAM and SPRINTARS.

	Models	Emission	Precipitation	RI[a]	Particle size[c]
**Default**	ECHAM-HAM	CMIP6 for BB and anthropogenic; AeroCom-II for biogenic emissions; Prescribed SOA productions; Online calculations for dust and sea salt	See ref (*65*)	1.85-0.71*i*	BB particles are emitted at 75 nm, with microphysical processes calculated by the modal scheme M7. Hygroscopic growth is based on the Kappa- Köhler theory (*65*).
	SPRINTARS	GFED4.1s for BB; CMIP6 for anthropogenic emission; GEIA for biogenic emissions; Online SOA production based on the oxidation of isoprene and terpenes; Online calculations for dust and sea salt	See ref (*55*)	1.75-0.44*i*	BB particles are emitted at 100 nm without growth of dry size. Hygroscopic growth refers to ref (*55*).
**Corrected**	ECHAM-HAM	BC and OA (both primary and secondary) emissions are corrected to our constrained results	Scaled by 3.3 in Africa[b]	1.85-0.3*i*	Increase the emitted size to 200 nm and scale the ambient size by 1.1
	SPRINTARS	BC and OA (both primary and secondary) emissions are corrected to our constrained results	Scaled by 0.3[b]	1.75-0.3*i*	100 nm emitted size with no hygroscopic growth

a. RI (refractive index) is shown for BC, and the RI for OA uses the model default values (see Table S1). The real parts of the BC refractive index in the corrected cases are the same as the default values as they have small impacts on the results.

b. The scaling factors based on modeled precipitation errors are applied to the wet removal directly.

c. The particle size refers to the number median radius.

Table S4. Comparison of the all-sky instantaneous direct radiative effect (unit: W m^{-2}) for the default and corrected simulations in the two global models. Data are presented as regional and seasonal average. The source and outflow regions are indicated in Fig. S10.

		Source region		Outflow region	
**Region**	**Model**	Default	Corrected	Default	Corrected
Amazon	ECHAM-HAM	-0.34	-4.35	0.49	-1.36
	SPRINTARS	-5.72	-6.70	-1.23	-1.80
Africa	ECHAM-HAM	0.04	-3.34	5.37	3.15
	SPRINTARS	-3.54	-3.56	-1.25	2.44

REFERENCES AND NOTES

1. V. Ramanathan, G. Carmichael, Global and regional climate changes due to black carbon. *Nat. Geosci.* **1**, 221–227 (2008).

2. P. V. Hobbs, J. S. Reid, R. A. Kotchenruther, R. J. Ferek, R. Weiss, Direct radiative forcing by smoke from biomass burning. *Science* **275**, 1777–1778 (1997).

3. L. Liu, Y. Cheng, S. Wang, C. Wei, M. L. Pöhlker, C. Pöhlker, P. Artaxo, M. Shrivastava, M. O. Andreae, U. Pöschl, H. Su, Impact of biomass burning aerosols on radiation, clouds, and precipitation over the Amazon: Relative importance of aerosol–cloud and aerosol–radiation interactions. *Atmos. Chem. Phys.* **20**, 13283–13301 (2020).

4. Y. J. Kaufman, I. Koren, Smoke and pollution aerosol effect on cloud cover. *Science* **313**, 655–658 (2006).

5. L. G. Jahl, T. A. Brubaker, M. J. Polen, L. G. Jahn, K. P. Cain, B. B. Bowers, W. D. Fahy, S. Graves, R. C. Sullivan, Atmospheric aging enhances the ice nucleation ability of biomass-burning aerosol. *Sci. Adv.* **7**, eabd3440 (2021).

6. J. C. Lin, T. Matsui, R. A. Pielke Sr., C. Kummerow, Effects of biomass-burning-derived aerosols on precipitation and clouds in the Amazon Basin: A satellite-based empirical study. *J. Geophys. Res.* **111**, D19204 (2006).

7. Ø. Hodnebrog, G. Myhre, P. M. Forster, J. Sillmann, B. H. Samset, Local biomass burning is a dominant cause of the observed precipitation reduction in southern Africa. *Nat. Commun.* **7**, 11236 (2016).

8. K. Ding, X. Huang, A. Ding, M. Wang, H. Su, V. M. Kerminen, T. Petäjä, Z. Tan, Z. Wang, D. Zhou, J. Sun, H. Liao, H. Wang, K. Carslaw, R. Wood, P. Zuidema, D. Rosenfeld, M. Kulmala, C. Fu, U. Pöschl, Y. Cheng, M. O. Andreae, Aerosol-boundary-layer-monsoon interactions amplify semi-direct effect of biomass smoke on low cloud formation in Southeast Asia. *Nat. Commun.* **12**, 6416 (2021).

9. F. Solmon, N. Elguindi, M. Mallet, C. Flamant, P. Formenti, West African monsoon precipitation impacted by the South Eastern Atlantic biomass burning aerosol outflow. *npj Clim. Atmos. Sci.* **4**, 54 (2021).

10. J. Redemann, R. Wood, P. Zuidema, S. J. Doherty, B. Luna, S. E. LeBlanc, M. S. Diamond, Y. Shinozuka, I. Y. Chang, R. Ueyama, L. Pfister, J. M. Ryoo, A. N. Dobracki, A. M. da Silva, K. M. Longo, M. S. Kacenelenbogen, C. J. Flynn, K. Pistone, N. M. Knox, S. J. Piketh, J. M. Haywood, P. Formenti, M. Mallet, P. Stier, A. S. Ackerman, S. E. Bauer, A. M. Fridlind, G. R. Carmichael, P. E. Saide, G. A. Ferrada, S. G. Howell, S. Freitag, B. Cairns, B. N. Holben, K. D. Knobelspiesse, S. Tanelli, T. S. L'Ecuyer, A. M. Dzambo, O. O. Sy, G. M. McFarquhar, M. R. Poellot, S. Gupta, J. R. O'Brien, A. Nenes, M. Kacarab, J. P. S. Wong, J. D. Small-Griswold, K. L. Thornhill, D. Noone, J. R. Podolske, K. S. Schmidt, P. Pilewskie, H. Chen, S. P. Cochrane, A. J. Sedlacek, T. J. Lang, E. Stith, M. Segal-Rozenhaimer, R. A. Ferrare, S. P. Burton, C. A. Hostetler, D. J. Diner, F. C. Seidel, S. E. Platnick, J. S. Myers, K. G. Meyer, D. A. Spangenberg, H. Maring, L. Gao, An overview of the ORACLES (ObseRvations of Aerosols above CLouds and their intEractionS) project: Aerosol–cloud–radiation interactions in the southeast Atlantic basin. *Atmos. Chem. Phys.* **21**, 1507–1563 (2021).

11. J. M. Haywood, S. J. Abel, P. A. Barrett, N. Bellouin, A. Blyth, K. N. Bower, M. Brooks, K. Carslaw, H. Che, H. Coe, M. I. Cotterell, I. Crawford, Z. Cui, N. Davies, B. Dingley, P. Field, P. Formenti, H. Gordon, M. de Graaf, R. Herbert, B. Johnson, A. C. Jones, J. M. Langridge, F. Malavelle, D. G. Partridge, F. Peers, J. Redemann, P. Stier, K. Szpek, J. W. Taylor, D. Watson-Parris, R. Wood, H. Wu, P. Zuidema, The CLoud–Aerosol–Radiation Interaction and Forcing: Year 2017 (CLARIFY-2017) measurement campaign. *Atmos. Chem. Phys.* **21**, 1049–1084 (2021).

12. S. T. Martin, P. Artaxo, L. A. T. Machado, A. O. Manzi, R. A. F. Souza, C. Schumacher, J. Wang, M. O. Andreae, H. M. J. Barbosa, J. Fan, G. Fisch, A. H. Goldstein, A. Guenther, J. L. Jimenez, U. Pöschl, M. A. Silva Dias, J. N. Smith, M. Wendisch, Introduction: Observations and modeling of the Green Ocean Amazon (GoAmazon2014/5), *Atmos. Chem. Phys.* **16**, 4785–4797 (2016).

13. G. Pereira, R. Siqueira, N. E. Rosário, K. L. Longo, S. R. Freitas, F. S. Cardozo, J. W. Kaiser, M. J. Wooster, Assessment of fire emission inventories during the South American Biomass Burning Analysis (SAMBBA) experiment. *Atmos. Chem. Phys.* **16**, 6961–6975 (2016).

14. H. Che, P. Stier, H. Gordon, D. Watson-Parris, L. Deaconu, Cloud adjustments dominate the overall negative aerosol radiative effects of biomass burning aerosols in UKESM1 climate model simulations over the south-eastern Atlantic. *Atmos. Chem. Phys.* **21**, 17–33 (2021).

15. S. J. Doherty, P. E. Saide, P. Zuidema, Y. Shinozuka, G. A. Ferrada, H. Gordon, M. Mallet, K. Meyer, D. Painemal, S. G. Howell, S. Freitag, A. Dobracki, J. R. Podolske, S. P. Burton, R. A. Ferrare, C. Howes, P. Nabat, G. R. Carmichael, A. da Silva, K. Pistone, I. Chang, L. Gao, R. Wood, J. Redemann, Modeled and observed properties related to the direct aerosol radiative effect of biomass burning aerosol over the southeastern Atlantic. *Atmos. Chem. Phys.* **22**, 1–46, (2022).

16. D. Chand, R. Wood, T. L. Anderson, S. K. Satheesh, R. J. Charlson, Satellite-derived direct radiative effect of aerosols dependent on cloud cover. *Nat. Geosci.* **2**, 181–184 (2009).

17. L. A. Lee, C. L. Reddington, K. S. Carslaw, On the relationship between aerosol model uncertainty and radiative forcing uncertainty. *Proc. Natl. Acad. Sci. U.S.A.* **113**, 5820–5827 (2016).

18. L. A. Regayre, J. S. Johnson, M. Yoshioka, K. J. Pringle, D. M. H. Sexton, B. B. B. Booth, L. A. Lee, N. Bellouin, K. S. Carslaw, Aerosol and physical atmosphere model parameters are both important sources of uncertainty in aerosol ERF. *Atmos. Chem. Phys.* **18**, 9975–10006 (2018).

19. M. Sand, B. H. Samset, G. Myhre, J. Gliß, S. E. Bauer, H. Bian, M. Chin, R. Checa-Garcia, P. Ginoux, Z. Kipling, A. Kirkevåg, H. Kokkola, P. Le Sager, M. T. Lund, H. Matsui, T. van Noije, D. J. L. Olivié, S. Remy, M. Schulz, P. Stier, C. W. Stjern, T. Takemura, K. Tsigaridis, S. G. Tsyro, D. Watson-Parris, Aerosol absorption in global models from AeroCom phase III. *Atmos. Chem. Phys.* **21**, 15929–15947 (2021).

20. M. Mallet, P. Nabat, B. Johnson, M. Michou, J. M. Haywood, C. Chen, O. Dubovik, Climate models generally underrepresent the warming by Central Africa biomass-burning aerosols over the Southeast Atlantic. *Sci. Adv.* **7**, eabg9998 (2021).

21. D. Koch, M. Schulz, S. Kinne, C. McNaughton, J. R. Spackman, Y. Balkanski, S. Bauer, T. Berntsen, T. C. Bond, O. Boucher, M. Chin, A. Clarke, N. De Luca, F. Dentener, T. Diehl, O. Dubovik, R. Easter, D. W. Fahey, J. Feichter, D. Fillmore, S. Freitag, S. Ghan, P. Ginoux, S. Gong, L. Horowitz, T. Iversen, A. Kirkevåg, Z. Klimont, Y. Kondo, M. Krol, X. Liu, R. Miller, V. Montanaro, N. Moteki, G. Myhre, J. E. Penner, J. Perlwitz, G. Pitari, S. Reddy, L. Sahu, H. Sakamoto, G. Schuster, J. P. Schwarz, Ø. Seland, P. Stier, N. Takegawa, T. Takemura, C. Textor, J. A. van Aardenne, Y. Zhao, Evaluation of black carbon estimations in global aerosol models. *Atmos. Chem. Phys.* **9**, 9001–9026 (2009).

22. K. Pistone, J. Redemann, S. Doherty, P. Zuidema, S. Burton, B. Cairns, S. Cochrane, R. Ferrare, C. Flynn, S. Freitag, S. G. Howell, M. Kacenelenbogen, S. LeBlanc, X. Liu, K. S. Schmidt, A. J. Sedlacek III, M. Segal-Rozenhaimer, Y. Shinozuka, S. Stamnes, B. van Diedenhoven, G. Van Harten, F. Xu, Intercomparison of biomass burning aerosol optical properties from in situ and remote-sensing instruments in ORACLES-2016. *Atmos. Chem. Phys.* **19**, 9181–9208 (2019).

23. C. Denjean, T. Bourrianne, F. Burnet, M. Mallet, N. Maury, A. Colomb, P. Dominutti, J. Brito, R. Dupuy, K. Sellegri, A. Schwarzenboeck, C. Flamant, P. Knippertz, Overview of aerosol optical properties over southern West Africa from DACCIWA aircraft measurements. *Atmos. Chem. Phys.* **20**, 4735–4756 (2020).

24. H. Brown, X. Liu, R. Pokhrel, S. Murphy, Z. Lu, R. Saleh, T. Mielonen, H. Kokkola, T. Bergman, G. Myhre, R. B. Skeie, D. Watson-Paris, P. Stier, B. Johnson, N. Bellouin, M. Schulz, V. Vakkari, J. P. Beukes, P. G. van Zyl, S. Liu, D. Chand, Biomass burning aerosols in most climate models are too absorbing. *Nat. Commun.* **12**, 277 (2021).

25. G. Schkolnik, D. Chand, A. Hoffer, M.O. Andreae, C. Erlick, E. Swietlicki, Y. Rudich, Constraining the density and complex refractive index of elemental and organic carbon in biomass burning aerosol using optical and chemical measurements. *Atmos. Environ.* **41**, 1107–1118 (2007).

26. B. H. Samset, G. Myhre, Vertical dependence of black carbon, sulphate and biomass burning aerosol radiative forcing. *Geophys. Res. Lett.* **38**, L24802 (2011).

27. E. Alonso-Blanco, A. I. Calvo, V. Pont, M. Mallet, R. Fraile, A. Castro, Impact of biomass burning on aerosol size distribution, aerosol optical properties and associated radiative forcing. *Aerosol Air Qual. Res.* **14**, 708–724 (2014).

28. Y. Wu, T. Cheng, X. Pan, L. Zheng, S. Shi, H. Liu, The role of biomass burning states in light absorption enhancement of carbonaceous aerosols. *Sci. Rep.* **10**, 12829 (2020).

29. B. H. Samset, C. W. Stjern, E. Andrews, R. A. Kahn, G. Myhre, M. Schulz, G. L. Schuster, Aerosol absorption: Progress towards global and regional constraints. *Curr. Clim. Change Rep.* **4**, 65–83 (2018).

30. M. K. Sporre, S. M. Blichner, R. Schrödner, I. H. H. Karset, T. K. Berntsen, T. van Noije, T. Bergman, D. O'Donnell, R. Makkonen, Large difference in aerosol radiative effects from BVOC-SOA treatment in three Earth system models. *Atmos. Chem. Phys.* **20**, 8953–8973 (2020).

31. Q. Zhong, N. Schutgens, G. R. van der Werf, T. van Noije, S. E. Bauer, K. Tsigaridis, T. Mielonen, R. Checa-Garcia, D. Neubauer, Z. Kipling, A. Kirkevåg, D. J. L. Olivié, H. Kokkola, H. Matsui, P. Ginoux, T. Takemura, P. Le Sager, S. Rémy, H. Bian, M. Chin, Using modelled relationships and satellite observations to attribute modelled aerosol biases over biomass burning regions. *Nat. Commun.* **13**, 5914 (2022).

32. J. H. Seinfeld, S. N. Pandis, *Atmospheric Chemistry and Physics: From Air Pollution to Climate Change* (Wiley, ed. 2, 2006), pp. 900–979.

33. A. C. Aiken, P. F. DeCarlo, J. H. Kroll, D. R. Worsnop, J. A. Huffman, K. S. Docherty, I. M. Ulbrich, C. Mohr, J. R. Kimmel, D. Sueper, Y. Sun, Q. Zhang, A. Trimborn, M. Northway, P. J. Ziemann, M. R. Canagaratna, T. B. Onasch, M. R. Alfarra, A. S. H. Prevot, J. Dommen, J. Duplissy, A. Metzger, U. Baltensperger, J. L. Jimenez, O/C and OM/OC ratios of primary, secondary, and ambient organic aerosols with high-resolution time-of-flight aerosol mass spectrometry. *Environ. Sci. Technol.* **42**, 4478–4485 (2008).

34. P. F. DeCarlo, I. M. Ulbrich, J. Crounse, B. de Foy, E. J. Dunlea, A. C. Aiken, D. Knapp, A. J. Weinheimer, T. Campos, P. O. Wennberg, J. L. Jimenez, Investigation of the sources and processing

of organic aerosol over the Central Mexican Plateau from aircraft measurements during MILAGRO. *Atmos. Chem. Phys.* **10**, 5257–5280 (2010).

35. X.-F. Huang, L.-Y. He, M. Hu, M. R. Canagaratna, J. H. Kroll, N. L. Ng, Y.-H. Zhang, Y. Lin, L. Xue, T.-L. Sun, X.-G. Liu, M. Shao, J. T. Jayne, D. R. Worsnop, Characterization of submicron aerosols at a rural site in Pearl River Delta of China using an aerodyne high-resolution aerosol mass spectrometer. *Atmos. Chem. Phys.* **11**, 1865–1877 (2011).

36. Z. Gong, Z. Lan, L. Xue, L. Zeng, L. He, X. Huang, Characterization of submicron aerosols in the urban outflow of the central Pearl River Delta region of China. *Front. Environ. Sci. Eng.* **6**, 725–733 (2012).

37. S. Saarikoski, S. Carbone, S. Decesari, L. Giulianelli, F. Angelini, M. Canagaratna, N. L. Ng, A. Trimborn, M. C. Facchini, S. Fuzzi, R. Hillamo, D. Worsnop, Chemical characterization of springtime submicrometer aerosol in Po Valley, Italy. *Atmos. Chem. Phys.* **12**, 8401–8421, (2012).

38. M. R. Canagaratna, J. L. Jimenez, J. H. Kroll, Q. Chen, S. H. Kessler, P. Massoli, L. Hildebrandt Ruiz, E. Fortner, L. R. Williams, K. R. Wilson, J. D. Surratt, N. M. Donahue, J. T. Jayne, D. R. Worsnop, Elemental ratio measurements of organic compounds using aerosol mass spectrometry: Characterization, improved calibration, and implications. *Atmos. Chem. Phys.* **15**, 253–272 (2015).

39. J. Zheng, M. Hu, Z. Du, D. Shang, Z. Gong, Y. Qin, J. Fang, F. Gu, M. Li, J. Peng, J. Li, Y. Zhang, X. Huang, L. He, Y. Wu, S. Guo, Influence of biomass burning from South Asia at a high-altitude mountain receptor site in China. *Atmos. Chem. Phys.* **17**, 6853–6864 (2017).

40. R. Ramo, E. Roteta, I. Bistinas, D. van Wees, A. Bastarrika, E. Chuvieco, G. R. van der Werf, African burned area and fire carbon emissions are strongly impacted by small fires undetected by coarse resolution satellite data. *Proc. Natl. Acad. Sci. U.S.A.* **118**, e2011160118 (2021).

41. S. S. de Sá, L. V. Rizzo, B. B. Palm, P. Campuzano-Jost, D. A. Day, L. D. Yee, R. Wernis, G. Isaacman-VanWertz, J. Brito, S. Carbone, Y. J. Liu, A. Sedlacek, S. Springston, A. H. Goldstein, H. M. J. Barbosa, M. L. Alexander, P. Artaxo, J. L. Jimenez, S. T. Martin, Contributions of biomass-burning, urban, and biogenic emissions to the concentrations and light-absorbing properties of

particulate matter in central Amazonia during the dry season. *Atmos. Chem. Phys.* **19**, 7973–8001 (2019).

42. P. I. Palmer, D. J. Jacob, A. M. Fiore, R. V. Martin, K. Chance, T. P. Kurosu, Mapping isoprene emissions over North America using formaldehyde column observations from space. *J. Geophys. Res. Atmos.* **108**, 4180 (2003).

43. J. P. Veefkind, K. F. Boersma, J. Wang, T. P. Kurosu, N. Krotkov, K. Chance, P. F. Levelt, Global satellite analysis of the relation between aerosols and short-lived trace gases. *Atmos. Chem. Phys.* **11**, 1255–1267 (2011).

44. Y. Zhang, R. Li, Q. Min, H. Bo, Y. Fu, Y. Wang, Z. Gao, The controlling factors of atmospheric formaldehyde (HCHO) in Amazon as seen from satellite. *Earth Space Sci.* **6**, 959–971 (2019).

45. J. Liao, T. F. Hanisco, G. M. Wolfe, J. St. Clair, J. L. Jimenez, P. Campuzano-Jost, B. A. Nault, A. Fried, E. A. Marais, G. G. Abad, K. Chance, H. T. Jethva, T. B. Ryerson, C. Warneke, A. Wisthaler, Towards a satellite formaldehyde—In situ hybrid estimate for organic aerosol abundance. *Atmos. Chem. Phys.* **19**, 2765–2785 (2019).

46. IPCC, 2021: Summary for policymakers, in *Climate Change 2021: The Physical Science Basis. Contribution of Working Group I to the Sixth Assessment Report of the Intergovernmental Panel on Climate Change* (Cambridge Univ. Press, 2021), pp. 3–32; https://doi.org/10.1017/9781009157896.001.

47. N. Schutgens, O. Dubovik, O. Hasekamp, O. Torres, H. Jethva, P. J. T. Leonard, P. Litvinov, J. Redemann, Y. Shinozuka, G. de Leeuw, S. Kinne, T. Popp, M. Schulz, P. Stier, AEROCOM and AEROSAT AAOD and SSA study—Part 1: Evaluation and intercomparison of satellite measurements. *Atmos. Chem. Phys.* **21**, 6895–6917 (2021).

48. L. Ma, T. Zhang, O. W. Frauenfeld, B. Ye, D. Yang, D. Qin, Evaluation of precipitation from the ERA-40, NCEP-1, and NCEP-2 Reanalyses and CMAP-1, CMAP-2, and GPCP-2 with ground-based measurements in China. *J. Geophys. Res.* **114**, D09105 (2009).

49. K. Chance, OMI/Aura Formaldehyde (HCHO) Total Column Daily L3 Weighted Mean Global 0.1deg Lat/Lon Grid V003, Greenbelt, MD, USA, Goddard Earth Sciences Data and Information Services Center (GES DISC) (2019); 10.5067/Aura/OMI/DATA3010 [accessed May 2022].

50. P. Guyon, B. Graham, G. C. Roberts, O. L. Mayol-Bracero, W. Maenhaut, P. Artaxo, M. O. Andreae, Sources of optically active aerosol particles over the Amazon forest. *Atmos. Environ.* **38**, 1039–1051 (2004).

51. M. Gyawali, W. P. Arnott, K. Lewis, H. Moosmüller, In situ aerosol optics in Reno, NV, USA during and after the summer 2008 California wildfires and the influence of absorbing and non-absorbing organic coatings on spectral light absorption. *Atmos. Chem. Phys.* **9**, 8007–8015 (2009).

52. D. A. Lack, J. M. Langridge, On the attribution of black and brown carbon light absorption using the Ångström exponent. *Atmos. Chem. Phys.* **13**, 10535–10543 (2013).

53. R. F. Adler, G. Gu, G. J. Huffman, Estimating climatological bias errors for the Global Precipitation Climatology Project (GPCP). *J. Appl. Meteorol. Climatol.* **51**, 84–99 (2012).

54. I. Tegen, D. Neubauer, S. Ferrachat, C. Siegenthaler-Le Drian, I. Bey, N. Schutgens, P. Stier, D. Watson-Parris, T. Stanelle, H. Schmidt, S. Rast, H. Kokkola, M. Schultz, S. Schroeder, N. Daskalakis, S. Barthel, B. Heinold, U. Lohmann, The global aerosol–climate model ECHAM6.3–HAM2.3—Part 1: Aerosol evaluation. *Geosci. Model Dev.* **12**, 1643–1677 (2019).

55. T. Takemura, T. Nozawa, S. Emori, T. Y. Nakajima, T. Nakajima, Simulation of climate response to aerosol direct and indirect effects with aerosol transport-radiation model. *J. Geophys. Res. Atmos.* **110,** D02202 (2005).

56. F. Dentener, S. Kinne, T. Bond, O. Boucher, J. Cofala, S. Generoso, P. Ginoux, S. Gong, J. J. Hoelzemann, A. Ito, L. Marelli, J. E. Penner, J.-P. Putaud, C. Textor, M. Schulz, G. R. van der Werf, J. Wilson, Emissions of primary aerosol and precursor gases in the years 2000 and 1750 prescribed data-sets for AeroCom. *Atmos. Chem. Phys.* **6,** 4321–4344 (2006).

57. Q. Zhong, N. Schutgens, G. van der Werf, T. van Noije, K. Tsigaridis, S. E. Bauer, T. Mielonen, A. Kirkevåg, Ø. Seland, H. Kokkola, R. Checa-Garcia, D. Neubauer, Z. Kipling, H. Matsui, P. Ginoux,

T. Takemura, P. Le Sager, S. Rémy, H. Bian, M. Chin, K. Zhang, J. Zhu, S. G. Tsyro, G. Curci, A. Protonotariou, B. Johnson, J. E. Penner, N. Bellouin, R. B. Skeie, G. Myhre, Satellite-based evaluation of AeroCom model bias in biomass burning regions. *Atmos. Chem. Phys.* **22**, 11009–11032 (2022).

58. K. Hungershoefer, K. Zeromskiene, Y. Iinuma, G. Helas, J. Trentmann, T. Trautmann, R. S. Parmar, A. Wiedensohler, M. O. Andreae, O. Schmid, Modelling the optical properties of fresh biomass burning aerosol produced in a smoke chamber: Results from the EFEU campaign. *Atmos. Chem. Phys.* **8**, 3427–3439 (2008).

59. O. Schmid, D. Chand, E. Karg, P. Guyon, G. P. Frank, E. Swietlicki, M. O. Andreae, Derivation of the density and refractive index of organic matter and elemental carbon from closure between physical and chemical aerosol properties. *Environ. Sci. Technol.* **43**, 1166–1172 (2009).

60. P. Chylek, J. E. Lee, D. E. Romonosky, F. Gallo, S. Lou, M. Shrivastava, C. M. Carrico, A. C. Aiken, M. K. Dubey, Mie scattering captures observed optical properties of ambient biomass burning plumes assuming uniform black, brown, and organic carbon mixtures. *J. Geophys. Res. Atmos.* **124**, 11406–11427 (2019).

61. E. J. T. Levin, G. R. McMeeking, C. M. Carrico, L. E. Mack, S. M. Kreidenweis, C. E. Wold, H. Moosmüller, W. P. Arnott, W. M. Hao, J. L. Collett Jr., W. C. Malm, Biomass burning smoke aerosol properties measured during Fire Laboratory at Missoula Experiments (FLAME). *J. Geophys. Res. Atmos.* **115**, D18210 (2010).

62. A. Siméon, F. Waquet, J.-C. Péré, F. Ducos, F. Thieuleux, F. Peers, S. Turquety, I. Chiapello, Combining POLDER-3 satellite observations and WRF-Chem numerical simulations to derive biomass burning aerosol properties over the southeast Atlantic region. *Atmos. Chem. Phys.* **21**, 17775–17805 (2021).

63. C. Mätzler, MATLAB functions for Mie scattering and absorption, version 2 (2002); https://boris.unibe.ch/146550/1/199.pdf.

64. Z. Meng, P. Yang, G. W. Kattawar, L. Bi, K. N. Liou, I. Laszlo, Single-scattering properties of tri-axial ellipsoidal mineral dust aerosols: A database for application to radiative transfer calculations. *J. Aerosol Sci.* **41**, 501–512 (2010).

65. K. Zhang, D. O'Donnell, J. Kazil, P. Stier, S. Kinne, U. Lohmann, S. Ferrachat, B. Croft, J. Quaas, H. Wan, S. Rast, J. Feichter, The global aerosol-climate model ECHAM-HAM, version 2: Sensitivity to improvements in process representations. *Atmos. Chem. Phys.* **12**, 8911–8949 (2012).

66. D. A. Lack, J. M. Langridge, R. Bahreini, C. D. Cappa, A. M. Middlebrook, J. P. Schwarz, Brown carbon and internal mixing in biomass burning particles. *Proc. Natl. Acad. Sci. U.S.A.* **109**, 14802–14807 (2012).

67. J. P. Schwarz, R. S. Gao, J. R. Spackman, L. A. Watts, D. S. Thomson, D. W. Fahey, T. B. Ryerson, J. Peischl, J. S. Holloway, M. Trainer, G. J. Frost, T. Baynard, D. A. Lack, J. A. de Gouw, C. Warneke, L. A. Del Negro, Measurement of the mixing state, mass, and optical size of individual black carbon particles in urban and biomass burning emissions. *Geophys. Res. Lett.* **35**, L13810 (2008).

68. X. Bi, G. Zhang, L. Li, X. Wang, M. Li, G. Sheng, J. Fu, Z. Zhou, Mixing state of biomass burning particles by single particle aerosol mass spectrometer in the urban area of PRD, China. *Atmos. Environ.* **45**, 3447–3453 (2011).

69. D. Liu, J. Whitehead, M. R. Alfarra, E. Reyes-Villegas, D. V. Spracklen, C. L. Reddington, S. Kong, P. I. Williams, Y.-C. Ting, S. Haslett, J. W. Taylor, M. J. Flynn, W. T. Morgan, G. McFiggans, H. Coe, J. D. Allan, Black-carbon absorption enhancement in the atmosphere determined by particle mixing state. *Nat. Geosci.* **10**, 184–188 (2017).

70. C. Denjean, J. Brito, Q. Libois, M. Mallet, T. Bourrianne, F. Burnet, R. Dupuy, C. Flamant, P. Knippertz, Unexpected biomass burning aerosol absorption enhancement explained by black carbon mixing state. *Geophys. Res. Lett.* **47**, e2020GL089055 (2020).

71. S. Kecorius, N. Ma, M. Teich, D. van Pinxteren, S. Zhang, J. Gröβ, G. Spindler, K. Müller, Y. Iinuma, M. Hu, H. Herrmann, A. Wiedensohler, Influence of biomass burning on mixing state of sub-micron aerosol particles in the North China Plain. *Atmos. Environ.* **164**, 259–269 (2017).

72. T. Stavrakou, J.-F. Müller, M. Bauwens, I. De Smedt, M. Van Roozendael, A. Guenther, M. Wild, X. Xia, Isoprene emissions over Asia 1979-2012: Impact of climate and land-use changes. *Atmos. Chem. Phys.* **14**, 4587–4605 (2014).

73. M. Bauwens, T. Stavrakou, J.-F. Müller, I. De Smedt, M. Van Roozendael, G. R. van der Werf, C. Wiedinmyer, J. W. Kaiser, K. Sindelarova, A. Guenther, Nine years of global hydrocarbon emissions based on source inversion of OMI formaldehyde observations. *Atmos. Chem. Phys.* **16**, 10133–10158 (2016).

74. J.-F. Müller, T. Stavrakou, S. Wallens, I. De Smedt, M. Van Roozendael, M. J. Potosnak, J. Rinne, B. Munger, A. Goldstein, A. B. Guenther, Global isoprene emissions estimated using MEGAN, ECMWF analyses and a detailed canopy environment model. *Atmos. Chem. Phys.* **8**, 1329–1341 (2008).

75. A. Kirkevåg, A. Grini, D. Olivié, Ø. Seland, K. Alterskjær, M. Hummel, I. H. H. Karset, A. Lewinschal, X. Liu, R. Makkonen, I. Bethke, J. Griesfeller, M. Schulz, T. Iversen, A production-tagged aerosol module for Earth system models, OsloAero5.3 - extensions and updates for CAM5. 3-Oslo. *Geosci. Model Dev.* **11,** 3945–3982 (2018).

76. X. Liu, R. C. Easter, S. J. Ghan, R. Zaveri, P. Rasch, X. Shi, J.-F. Lamarque, A. Gettelman, H. Morrison, F. Vitt, A. Conley, S. Park, R. Neale, C. Hannay, A. M. L. Ekman, P. Hess, N. Mahowald, W. Collins, M. J. Iacono, C. S. Bretherton, M. G. Flanner, D. Mitchell, Toward a minimal representation of aerosols in climate models: Description and evaluation in the Community Atmosphere Model CAM5. *Geosci. Model Dev.* **5**, 709–739 (2012).

77. H. Matsui, Development of a global aerosol model using a two-dimensional sectional method: 1. Model design. *J. Adv. Model. Earth Syst.* **9,** 1921–1947 (2017).

78. T. van Noije, T. Bergman, P. Le Sager, D. O'Donnell, R. Makkonen, M. Gonçalves-Ageitos, R. Döscher, U. Fladrich, J. von Hardenberg, J.-P. Keskinen, H. Korhonen, A. Laakso, S. Myriokefalitakis, P. Ollinaho, C. P. García-Pando, T. Reerink, R. Schrödner, K. Wyser, S. Yang, EC-Earth3-AerChem: A global climate model with interactive aerosols and atmospheric chemistry participating in CMIP6. *Geosci. Model Dev.* **14,** 5637–5668 (2021).

79. H. Kokkola, T. Kühn, A. Laakso, T. Bergman, K. E. J. Lehtinen, T. Mielonen, A. Arola, S. Stadtler, H. Korhonen, S. Ferrachat, U. Lohmann, D. Neubauer, I. Tegen, C. S.-L. Drian, M. G. Schultz, I. Bey, P. Stier, N. Daskalakis, C. L. Heald, S. Romakkaniemi, SALSA2.0: The sectional aerosol module of the aerosol-chemistry-climate model ECHAM6.3.0-HAM2.3-MOZ1.0. *Geosci. Model Dev.* **11,** 3833–3863 (2018).

80. S. Rémy, Z. Kipling, V. Huijnen, J. Flemming, P. Nabat, M. Michou, M. Ades, R. Engelen, V.-H. Peuch, Description and evaluation of the tropospheric aerosol scheme in the European Centre for Medium-Range Weather Forecasts (ECMWF) Integrated Forecasting System (IFS-AER, cycle 45R1). *Geosci. Model Dev.* **12,** 4627–4659 (2019).

81. P. Colarco, A. da Silva, M. Chin, T. Diehl, Online simulations of global aerosol distributions in the NASA GEOS-4 model and comparisons to satellite and ground-based aerosol optical depth. *J. Geophys. Res. Atmos.* **115,** D14207 (2010).

82. M. Zhao, J.-C. Golaz, I. M. Held, H. Guo, V. Balaji, R. Benson, J.-H. Chen, X. Chen, L. J. Donner, J. P. Dunne, K. Dunne, J. Durachta, S.-M. Fan, S. M. Freidenreich, S. T. Garner, P. Ginoux, L. M. Harris, L. W. Horowitz, J. P. Krasting, A. R. Langenhorst, Z. Liang, P. Lin, S.-J. Lin, S. L. Malyshev, E. Mason, P. C. D. Milly, Y. Ming, V. Naik, F. Paulot, D. Paynter, P. Phillipps, A. Radhakrishnan, V. Ramaswamy, T. Robinson, D. Schwarzkopf, C. J. Seman, E. Shevliakova, Z. Shen, H. Shin, L. G. Silvers, J. R. Wilson, M. Winton, A. T. Wittenberg, B. Wyman, B. Xiang, The GFDL Global Atmosphere and Land Model AM4.0/LM4.0: 2. Model description, sensitivity studies, and tuning strategies. *J. Adv. Model. Earth Sys.* **10,** 735–769 (2018).

83. S. E. Bauer, K. Tsigaridis, G. Faluvegi, M. Kelley, K. K. Lo, R. L. Miller, L. Nazarenko, G. A. Schmidt, J. Wu, Historical (1850–2014) aerosol evolution and role on climate forcing using the GISS ModelE2.1 contribution to CMIP6. *J. Adv. Model. Earth Syst.* **8,** e2019MS001978 (2020).

84. M. Schulz, A. Cozic, S. Szopa, LMDzT-INCA dust forecast model developments and associated validation efforts. *IOP Conf. Ser. Earth Environ. Sci.* **7,** 12014 (2009).

85. R. Wang, Y. Balkanski, O. Boucher, P. Ciais, G. L. Schuster, F. Chevallier, B. H. Samset, J. Liu, S. Piao, M. Valari, S. Tao, Estimation of global black carbon direct radiative forcing and its uncertainty constrained by observations. *J. Geophys. Res. Atmos.* **121**, 5948–5971 (2016).

86. T. P. C. van Noije, P. Le Sager, A. J. Segers, P. F. J. van Velthoven, M. C. Krol, W. Hazeleger, A. G. Williams, S. D. Chambers, Simulation of tropospheric chemistry and aerosols with the climate model EC-Earth. *Geosci. Model Dev.* **7,** 2435–2475 (2014).

87. M. O. Andreae, E. V. Browell, M. Garstang, G. L. Gregory, R. C. Harriss, G. F. Hill, D. J. Jacob, M. C. Pereira, G. W. Sachse, A. W. Setzer, P. L. Silva Dias, R. W. Talbot, A. L. Torres, S. C. Wofsy, Biomass-burning emissions and associated haze layers over Amazonia. *J. Geophys. Res.* **93**, 1509–1527 (1988).

88. D. E. Ward, C. C. Hardy, Smoke emissions from wildland fires. *Environ. Int.* **17**, 117–134 (1991).

89. R. J. Ferek, J. S. Reid, P. V. Hobbs, D. R. Blake, C. Liousse, Emission factors of hydrocarbons, halocarbons, trace gases and particles from biomass burning in Brazil. *J. Geophys. Res.* **103**, 32107–32118 (1998).

90. M. A. Yamasoe, P. Artaxo, A. H. Miguel, A. G Allen, Chemical composition of aerosol particles from direct emissions of vegetation fires in the Amazon Basin: Water-soluble species and trace elements. *Atmos. Environ.* **34**, 1641–1653 (2000).

91. M. O. Andreae, P. Merlet, Emission of trace gases and aerosols from biomass burning. *Global Biogeochem. Cy.* **15**, 955–966 (2001).

92. P. Guyon, G. P. Frank, M. Welling, D. Chand, P. Artaxo, L. Rizzo, G. Nishioka, O. Kolle, H. Fritsch, M. A. F Silva Dias, L. V. Gatti, A. M. Cordova, M. O. Andreae, Airborne measurements of trace gas and aerosol particle emissions from biomass burning in Amazonia. *Atmos. Chem. Phys.* **5**, 2989–3002 (2005).

93. R. J. Yokelson, J. D. Crounse, P. F. DeCarlo, T. Karl, S. Urbanski, E. Atlas, T. Campos, Y. Shinozuka, V. Kapustin, A. D. Clarke, A. Weinheimer, D. J. Knapp, D. D. Montzka, J. Holloway, P. Weibring, F. Flocke, W. Zheng, D. Toohey, P. O. Wennberg, C. Wiedinmyer, L. Mauldin, A. Fried, D. Richter, J. Walega, J. L. Jimenez, K. Adachi, P. R. Buseck, S. R. Hall, R. Shetter, Emissions from biomass burning in the Yucatan. *Atmos. Chem. Phys.* **9**, 5785–5812 (2009).

94. S. K. Akagi, R. J. Yokelson, C. Wiedinmyer, M. J. Alvarado, J. S. Reid, T. Karl, J. D. Crounse, P. O. Wennberg, Emission factors for open and domestic biomass burning for use in atmospheric models. *Atmos. Chem. Phys.* **11**, 4039–4072 (2011).

95. A. K. Hodgson, W. T. Morgan, S. O'Shea, S. Bauguitte, J. D. Allan, E. Darbyshire, M. J. Flynn, D. Liu, J. Lee, B. Johnson, J. M. Haywood, K. M. Longo, P. E. Artaxo, H. Coe, Near-field emission profiling of tropical forest and Cerrado fires in Brazil during SAMBBA 2012. *Atmos. Chem. Phys.* **18**, 5619–5638 (2018).

96. M. L. Pöhlker, F. Ditas, J. Saturno, T. Klimach, I. H. de Angelis, A. C. Araùjo, J. Brito, S. Carbone, Y. Cheng, X. Chi, R. Ditz, S. S. Gunthe, B. A. Holanda, K. Kandler, J. Kesselmeier, T. Könemann, O. O. Krüger, J. V. Lavrič, S. T. Martin, E. Mikhailov, D. Moran-Zuloaga, L. V. Rizzo, D. Rose, H. Su, R. Thalman, D. Walter, J. Wang, S. Wolff, H. M. J. Barbosa, P. Artaxo, M. O. Andreae, U. Pöschl, C. Pöhlker, Long-term observations of cloud condensation nuclei over the Amazon rain forest—Part 2: Variability and characteristics of biomass burning, long-range transport, and pristine rain forest aerosols. *Atmos. Chem. Phys.* **18**, 10289–10331 (2018).

97. M. O. Andreae, Emission of trace gases and aerosols from biomass burning—An updated assessment. *Atmos. Chem. Phys.* **19**, 8523–8546 (2019).

98. D. E. Ward, C. C. Hardy, *Emissions from prescribed burning of chaparral*, www.frames.gov/documents/smoke/serdp/ward_hardy_1989b.pdf (1989).

99. H. Cachier, C. Liousse, P. Buat-Menard, A. Gaudichet, Particulate content of savanna fire emissions. *J. Atmos. Chem.* **22**, 123–148 (1995).

100. F. Echalar, A. Gaudichet, H. Cachier, P. Artaxo, Aerosol emissions by tropical forest and savanna biomass burning: Characteristic trace elements and fluxes. *Geophys. Res. Lett.* **22**, 3039–3042 (1995).

101. M. O. Andreae, E. Atlas, H. Cachier, W. R. Cofer, III, G. W. Harris, G. Helas, R. Koppmann, J.-P. Lacaux, D. E. Ward, Trace gas and aerosol emissions from savanna fires, in Biomass Burning and Global Change, edited by J.S. Levine, 278–295, MIT Press, Cambridge, Mass. (1996).

102. M. O. Andreae, T. W. Andreae, H. Annegarn, J. Beer, H. Cachier, P. Le Canut, W. Elbert, W. Maenhaut, I. Salma, F. G. Wienhold, T. Zenker, Airborne studies of aerosol emissions from savanna fires in southern Africa: 2 Aerosol chemical composition. *J. Geophys. Res.* **103**, 32119–32128 (1998).

103. P. Formenti, W. Elbert, W. Maenhaut, J. Haywood, S. Osborne, M. O. Andreae, Inorganic and carbonaceous aerosols during the Southern African Regional Science Initiative (SAFARI 2000) experiment: Chemical characteristics, physical properties, and emission data for smoke from African biomass burning. *J. Geophys. Res.* **108**, 8488 (2003).

104. P. Sinha, P. V. Hobbs, R. J. Yokelson, I. T. Bertschi, D. R. Blake, I. J. Simpson, S. Gao, T. W. Kirchstetter, T. Novakov, Emissions of trace gases and particles from savanna fires in southern Africa. *J. Geophys. Res.* **108**, 8487 (2003).

105. P. Sinha, P. V. Hobbs, R. J. Yokelson, D. R. Blake, S. Gao, T. W. Kirchstetter, Emissions from miombo woodland and dambo grassland savanna fires. *J. Geophys. Res. Atmos.* **109**, D11305 (2004).

106. G. Capes, B. Johnson, G. McFiggans, P. I. Williams, J. Haywood, H. Coe, Aging of biomass burning aerosols over West Africa: Aircraft measurements of chemical composition, microphysical properties, and emission ratios. *J. Geophys. Res.* **113**, D00C15 (2008).

107. S. K. Akagi, R. J. Yokelson, I. R. Burling, S. Meinardi, I. Simpson, D. R. Blake, G. R. McMeeking, A. Sullivan, T. Lee, S. Kreidenweis, S. Urbanski, J. Reardon, D. W. T. Griffith, T. J. Johnson, D. R. Weise, Measurements of reactive trace gases and variable O3 formation rates in some South Carolina biomass burning plumes. *Atmos. Chem. Phys.* **13**, 1141–1165 (2013).

108. A. A. May, G. R. McMeeking, T. Lee, J. W. Taylor, J. S. Craven, I. Burling, A. P. Sullivan, S. Akagi, J. L. Collett Jr., M. Flynn, H. Coe, S. P. Urbanski, J. H. Seinfeld, R. J. Yokelson, S. M. Kreidenweis, Aerosol emissions from prescribed fires in the United States: A synthesis of laboratory and aircraft measurements. *J. Geophys. Res. Atmos.* **119**, 11826–11849 (2014).

109. S. J. Lawson, M. D. Keywood, I. E. Galbally, J. L. Gras, J. M. Cainey, M. E. Cope, P. B. Krummel, P. J. Fraser, L. P. Steele, S. T. Bentley, C. P. Meyer, Z. Ristovski, A. H. Goldstein, Biomass burning emissions of trace gases and particles in marine air at Cape Grim, Tasmania, *Atmos. Chem. Phys.* **15**, 13393–13411 (2015).

110. A. L. Holder, G. S. W. Hagler, J. Aurell, M. D. Hays, B. K. Gullett, Particulate matter and black carbon optical properties and emission factors from prescribed fires in the southeastern United States. *J. Geophys. Res. Atmos.* **121**, 3465–3483 (2016).

111. M. Desservettaz, C. Paton-Walsh, D. W. T. Griffith, G. Kettlewell, M. D. Keywood, M. V. Vanderschoot, J. Ward, M. D. Mallet, A. Milic, B. Miljevic, Z. D. Ristovski, D. Howard, G. C. Edwards, B. Atkinson, Emission factors of trace gases and particles from tropical savanna fires in Australia. *J. Geophys. Res. Atmos.* **122**, 6059–6074 (2017).
